# Superhydrophobic‐Substrate‐Assisted Construction of Free‐Standing Microcavity‐Patterned Conducting Polymer Films

**DOI:** 10.1002/advs.202100949

**Published:** 2021-07-10

**Authors:** Yupeng Chen, Zhongpeng Zhu, Xiangyu Jiang, Lei Jiang

**Affiliations:** ^1^ Key Laboratory of Bio‐Inspired Smart Interfacial Science and Technology of Ministry of Education School of Chemistry Beihang University Beijing 100191 P. R. China; ^2^ CAS Key Laboratory of Bio‐Inspired Materials and Interfacial Science CAS Center for Excellence in Nanoscience Technical Institute of Physics and Chemistry Chinese Academy of Sciences Beijing 100190 P. R. China; ^3^ University of Chinese Academy of Sciences Beijing 100049 P. R. China; ^4^ School of Future Technology University of Chinese Academy of Sciences Beijing 101407 China

**Keywords:** electrochemical polymerization, epitaxial growth, free‐standing, microcavity‐patterned conducting polymer films, superhydrophobic micropillars

## Abstract

Patterned conducting polymer films with unique structures have promising prospects for application in various fields, such as actuation, water purification, sensing, and bioelectronics. However, their practical application is hindered because of the limitations of existing construction methods. Herein, a strategy is proposed for the superhydrophobic‐substrate‐assisted construction of free‐standing 3D microcavity‐patterned conducting polymer films (McPCPFs) at micrometer resolution. Easy‐peeling and nondestructive transfer properties are achieved through electrochemical polymerization along the solid/liquid/gas triphase interface on micropillar‐structured substrates. The effects of the wettability and geometrical parameters of the substrates on the construction of McPCPFs are systematically investigated in addition to the evolution of the epitaxial growth along the triphase interface at different polymerization times. The McPCPFs can be easily peeled from superhydrophobic surfaces using ethanol because of weak adhesion and nondestructively transferred to various substrates taking advantage of the capillarity. Furthermore, sensitive light‐driven McPCPF locomotion on organic liquid surfaces is demonstrated. Ultimately, a facile strategy for the construction of free‐standing 3D microstructure‐patterned conducting polymer films is proposed, which can improve productivity and applicability of the films in different fields and expand the application scope of superwettable interfaces.

## Graphical abstract

Superhydrophobic‐substrate‐assisted construction of free‐standing microcavity‐patterned conducting polymer films (McPCPFs) at micrometer resolution with easy‐peeling and nondestructive transfer properties is achieved through electrochemical polymerization along the triphase interface. The effects of the wettability and geometrical parameters of micropillar‐structured substrates on the construction of McPCPFs are systematically investigated, followed by an exploration of the epitaxial growth and a demonstration in light‐driven locomotion.

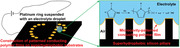

## 1 Introduction

Patterned conducting polymer films with unique structures are endowed with enhanced physicochemical properties, allowing their broad application in the fields of actuation,^[^ ^1^ ^]^ water purification,^[^ ^2^ ^]^ sensing,^[^ ^3^ ^]^ and bioelectronics.^[^ ^4^ ^]^ For instance, the aligned microtubule layers of polypyrrole (PPy) bilayer actuators can efficiently capture more polystyrene (PS) particles with a smaller size than a compact layer because of the high density of traps between the microtubules.^[^ ^1b^ ^]^ In addition, 3D PPy origami materials exhibit high solar‐thermal energy conversion efficiency in contrast to their planar counterparts, because of the increased area for vapor dissipation.^[^ ^2a^ ^]^ Furthermore, highly oriented single‐crystal PPy nanotubes with a low wall thickness exhibit an ultralow ammonia gas detection limit (≈0.05 ppb).^[^ ^5^ ^]^ Therefore, the development of effective preparation methods for the fabrication of patterned conducting polymer films characterized by specific micro/nanostructures is a meaningful pursuit. Generally, conducting polymer films consisting of nanowires, nanotubes, and inverse opals can be fabricated through chemical or electrochemical polymerization on hard (e.g., anodic aluminum oxide^[^ ^6^ ^]^) or soft (e.g., PS^[^ ^7^ ^]^) templates after further dissolution in inorganic acid/base solutions or organic reagents. However, because this fabrication method involves wasteful sacrificing of templates, it results in low productivity, thereby limiting its practical application. To date, various printing methods, such as microcontact printing,^[^ ^8^ ^]^ inkjet printing,^[^ ^9^ ^]^ dip‐pen nanolithography,^[^ ^10^ ^]^ scanning probe‐based lithography,^[^ ^11^ ^]^ the scanned pipette technique,^[^ ^12^ ^]^ and 3D printing^[^ ^13^ ^]^ have been developed to improve the productivity of the fabrication method for patterned conducting polymer films with unique structures. For instance, microcontact printing is used to fabricate patterned conducting polymer films at the microscale or sub‐microscale on conductive or nonconductive substrates through the use of patterned stamps, such as polydimethylsiloxane (PDMS)^[^ ^8a,b^ ^]^ and hydrogel stamps.^[^ ^8c^ ^]^ Inkjet printing can deliver polymer inks^[^ ^9a,b^ ^]^ to specific substrates or oxidant inks^[^ ^9c^ ^]^ to conducting polymer coatings. This method is usually employed to form patterned conducting polymer films at the microscale, which requires good dispersion of the polymers in the inks. For dip‐pen nanolithography, the polymer ink can be delivered to the target substrates using atomic force microscope probes to obtain 2D nanoscale patterns.^[^ ^10^ ^]^ Scanning probe‐based lithography is capable of delivering polymer precursor solutions to substrates, and nanoscale patterns can be fabricated after solid‐state oxidation crosslinking^[^ ^11a^ ^]^ or electrochemical polymerization.^[^ ^11b^ ^]^ The abovementioned printing methods are typically employed for preparing 2D patterned conducting polymer films at the microscale, sub‐microscale, and nanoscale, which can hardly mediate finer 3D structures. Recently, the scanned pipette technique has been applied to construct 3D patterned conducting polymer films at the microscale and nanoscale through polymerization^[^ ^12a^ ^–^ ^c^ ^]^ or evaporation^[^ ^12d,e^ ^]^ after delivering polymer precursor solutions or polymer solutions to the substrates. However, this technique involves complex processes and the prepared patterns are often independent of each other. In comparison, 3D printing is a fast approach for fabricating 3D patterned conducting polymer films using polymer inks.^[^ ^13^ ^]^ However, this method requires the polymer inks with good rheological properties, and the printing resolution ratio needs to be improved. Thus, there is an urgent need to develop a facile method for the fabrication of 3D patterned conducting polymer films with fine structures to allow their application in different fields.

At present, superwettable (e.g., superamphiphilic and superamphiphobic) interfaces provide an effective platform for constructing flat films and structured patterns based on chemical reactions or assembly.^[^ ^14^ ^]^ Uniformly smooth conducting polymer films, such as PPy films are obtained on both superamphiphilic silicon surfaces^[^ ^15^ ^]^ in air and superoleophilic organogel surfaces^[^ ^16^ ^]^ under water through chemical polymerization. In addition, inorganic BaTiO_3_ superlattices with different shapes can be formed on superhydrophilic micropillars through assembly.^[^ ^17^ ^]^ Superhydrophobic micropillars can be employed to fabricate patterned metals (e.g., Au microflowers^[^ ^18^ ^]^) and inorganic materials (e.g., metal sulfide microflowers^[^ ^19^ ^]^) with unique structures through electrodeposition or chemical deposition, as well as patterned inorganic crystals (e.g., NaCl crystals^[^ ^20^ ^]^) and organic micro/nanowires (e.g., spider silk nanowires^[^ ^21^ ^]^) through assembly. Moreover, micropillars with asymmetric wettability, such as hydrophilic sides and hydrophobic tops, and hydrophilic tops and hydrophobic sides, can be used to assemble inorganic crystal nanowires^[^ ^22^ ^]^ and organic crystal microrings,^[^ ^23^ ^]^ respectively. Recently, it has been reported that Au‐areole patterns with spiky structures have been electrodeposited on patterned superhydrophilic–superhydrophobic silicon surfaces for efficient Raman detection.^[^ ^24^ ^]^ Furthermore, taking advantage of the adjustable wettability of Au‐coated micropillars, uniform 3D façades consisting of semiconducting materials were assembled to enhance omnidirectional light detection.^[^ ^25^ ^]^ Therefore, it would be useful to develop structured interfaces with superwettability in order to explore their application in the fabrication of patterned conducting polymer films with fine 3D structures.

Herein, we propose a strategy for the superhydrophobic‐substrate‐assisted construction of free‐standing 3D microcavity‐patterned conducting polymer films (McPCPFs). First, the effects of the wettability and geometrical parameters (e.g., diameter, distance, shape, and arrangement) of micropillar‐structured substrates on the construction of McPCPFs are systematically investigated. Second, the epitaxial growth of patterned conducting polymer films along the solid/liquid/gas triphase interface at different electrochemical polymerization times is explored and the generality of this strategy is discussed. Furthermore, these McPCPFs exhibit easy‐peeling property due to the weaker adhesion and nondestructive transfer property to various substrates with different wettability taking advantage of the capillarity. The challenges to the preparation of larger McPCPFs are also discussed. Finally, the enhancement of light‐driven locomotion on organic liquid surfaces through the use of McPCPFs owing to effective photothermal conversion is investigated. Thus, a facile strategy for the construction of free‐standing 3D microstructure‐patterned conducting polymer films at micrometer resolution with easy‐peeling and nondestructive transfer properties is achieved. This strategy has great potential not only for improving the productivity of patterned conducting polymer films with fine structures and promoting their applications but also for expanding the application scope of superwettable interfaces.

## 2 Results and Discussion

### 2.1 Construction of McPCPFs

**Figure 1:**
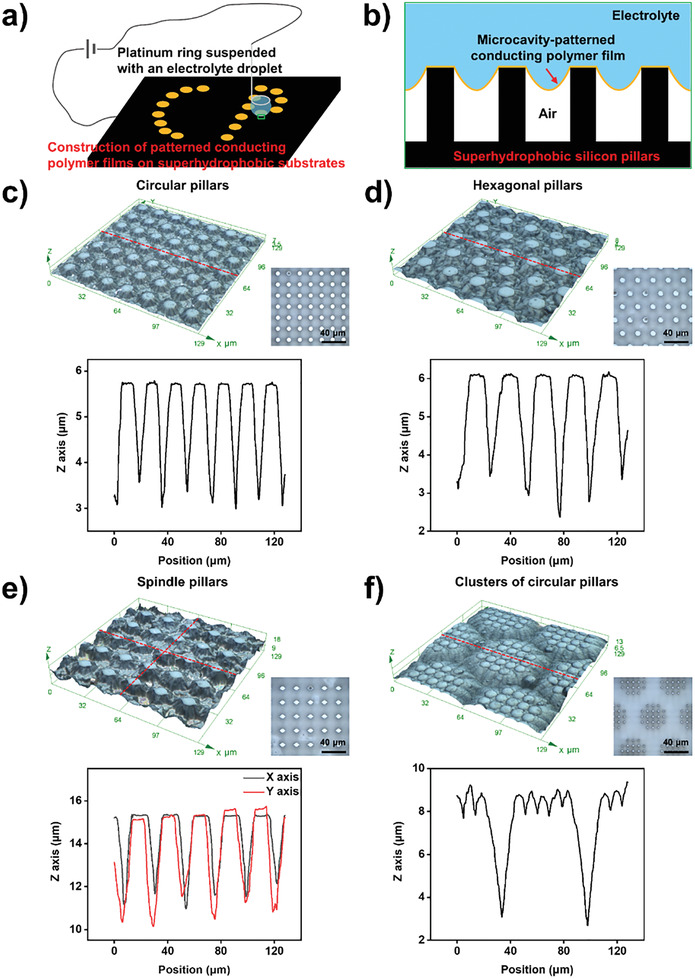
Superhydrophobic‐substrate‐assisted construction of 3D microcavity‐patterned conducting polymer films (McPCPFs). a) Schematic illustration of the experimental setup consisting of a platinum ring as a counter electrode, an electrolyte droplet containing conducting polymer monomers and electrolyte, a superhydrophobic micropillar‐structured substrate as a working electrode, as well as a Keithley 2611B power source. b) Enlarged view of the McPCPFs (i.e., PPy) formed along the triphase interface in the green box in a). 3D laser‐scanning microscopic images of McPCPFs (top left), plane microscopic images of micropillar‐structured substrates (top right), and the corresponding profiles (bottom) along the red dotted lines of McPCPFs with different base shapes in different arrangements, such as a circular base in a square arrangement c), a hexagonal base in a hexagonal arrangement d), an anisotropic spindle base in a square arrangement e) and a hierarchical circular base in a hexagonal arrangement f).

In this study, we proposed a strategy for the superhydrophobic‐substrate‐assisted construction of McPCPFs. The experimental setup consisted of a platinum ring as a counter electrode, an electrolyte droplet containing conducting polymer monomers (i.e., pyrrole) and electrolyte (i.e., dodecylbenzene sulfonic acid (HDBS)), a superhydrophobic micropillar‐structured substrate as a working electrode, and a Keithley 2611B power source (**Figure** **1**a; and Figure S1a, Supporting Information). Taking a substrate with a square arrangement of circular micropillars, for example, it exhibited superhydrophobicity with a water droplet contact angle of 154.8 ± 0.6° after decorating a monolayer of heptadecafluoro‐decyltrimethoxysilane (FAS) (Figure S1b, Supporting Information), because of the formation of a solid/liquid/gas triphase interface comprising the micropillars, air, and the water droplet as shown in the green box in the inset optical image in Figure S1b (Supporting Information). Next, a 20 µL electrolyte droplet containing 0.24 m pyrrole and 0.01 m HDBS was slowly added to the platinum ring using a pipette to make contact with the superhydrophobic substrate below. Then, the experimental setup was powered with a constant current of 20 µA and McPCPFs with a square arrangement of circular microcavities can be polymerized along the triphase interface (Figure 1b; and Figure S1c,d, Supporting Information). In addition, McPCPFs can be polymerized at different locations on the substrate by repositioning the platinum ring.

Moreover, the profiles of the McPCPFs depend on the geometrical parameters of the micropillar‐structured substrates. Thus, by choosing substrates with different shaped pillars in different arrangements, various McPCPFs with different microcavity base shapes in different arrangements can be constructed (Figure 1c–f). In detail, 3D McPCPFs with a circular base in a square arrangement and a hexagonal base in a hexagonal arrangement were obtained on micropillar‐structured substrates with a square arrangement of circular micropillars and a hexagonal arrangement of hexagonal micropillars, respectively. In addition, McPCPFs with an anisotropic spindle base in a square arrangement can be constructed using the micropillar‐structured substrates with a square arrangement of spindle micropillars, as shown by the distinct profiles along the *X*‐axis and *Y*‐axis in Figure 1e. What's more, McPCPFs with a hierarchical circular base in a hexagonal arrangement were also fabricated on micropillar‐structured substrates with a hexagonal arrangement of circular micropillar clusters (Figure 1f). Therefore, a facile strategy is proposed to construct McPCPFs through electrochemical polymerization along the solid/liquid/gas triphase interface.

### 2.2 Effects of Wettability and Geometrical Parameters of Micropillar‐Structured Substrates

**Figure 2:**
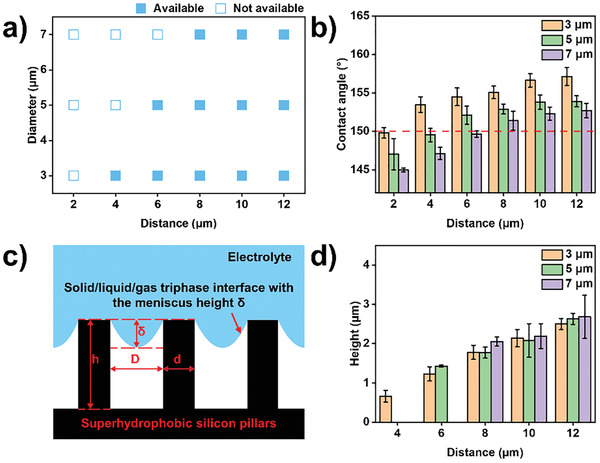
Effects of the wettability and geometrical parameters of micropillar‐structured substrates on constructing McPCPFs. a) The dependence of McPCPFs on the geometrical parameters of micropillar‐structured substrates. The solid and hollow blue squares represent the situations which are available and not available for constructing McPCPFs, respectively. b) The dependence of the wettability of micropillar‐structured substrates on the geometrical parameters. The red dotted line represents the boundary of superhydrophobicity. c) Schematic illustration of the solid/liquid/gas triphase interface with the meniscus height *δ* consisting of the micropillars, air, and the electrolyte droplet. The micropillar diameter, distance, and height are represented by *d*, *D*, and *h*, respectively. d) The dependence of the height of the microcavities determined by the meniscus height *δ* on the micropillar distance *D*.

To systematically investigate the effects of the wettability and geometrical parameters of micropillar‐structured substrates on the construction of McPCPFs, a series of substrates with a hexagonal arrangement of circular micropillars were employed after the silanization treatment. The dependence of the McPCPFs on the geometrical parameters (e.g., diameter and distance) is conveyed through **Figure** **2**a. Notably, McPCPFs were not formed when substrates with the following diameter/distance values (µm): 3/2, 5/2, 5/4, 7/2, 7/4, and 7/6 were used (Figures S2, S3, and S4, Supporting Information). Upon analyzing the wettability of these substrates, the main reason for failure to construct the McPCPFs is the absence of the solid/liquid/gas triphase interface (Figure 2b; and Figure S5, Supporting Information). As these substrates have large diameters and small distances, the fractional flat geometrical areas of the solid/liquid and gas/liquid interfaces under the droplet are too large and small, respectively. In these cases, the substrates exhibited lower water droplet contact angles (<150°) and could not prepare McPCPFs because the surfactant electrolyte droplets with lower surface tension tended to penetrate and fill the micropillar gaps to form the Wenzel state as shown in Figure S5 (Supporting Information). What's more, the computing of critical angles for the substrates can provide a guidance for the predesign of the micropillar‐structured substrates which were favorable to construct McPCPFs (Figure S6 and Tables S1–S3, Supporting Information).^[^ ^26^ ^]^ The substrates with higher water droplet contact angles (>150°) were still able to support the electrolyte droplets, forming a triphase interface with a meniscus height *δ* (*δ* < *h* at the Cassie state ) (Figure 2c). Furthermore, the water droplet contact angles of these substrates increase with an increase in the micropillar distance for micropillars with different diameters, which can be illustrated through the following Cassie equations^[^ ^27^ ^]^

(1) $$\cos\theta =f_{\mathrm{SL}}\left(\cos\theta_{0}+1\right)-1$$

(2) $$f_{\mathrm{SL}}=\pi d^{2}/2\sqrt{3}{\left(D+d\right)}^{2}$$

where *θ* and *θ*_0_ are the contact angles for micropillar structured and flat substrates, respectively, *f*_SL_ is the fractional flat geometrical area of the solid/liquid interface under a droplet, and *D* and *d* are the micropillar distance and diameter, respectively. By calculating *f*_SL_ for the micropillar‐structured substrates used in Figure 2a, it is found that when *f*_SL_ is 3.63–19.75%, the McPCPFs can be constructed successfully. Although, an increase in *D* will lead to a decrease in *f*_SL_ and an increase of the water droplet contact angle. *D* should satisfy *D* ^2^/*R* < *h* (*R* is the droplet radius and *h* is the micropillar height), where the maximum meniscus height is smaller than the micropillar height. When the maximum meniscus height is larger than the micropillar height (*D* ^2^/*R* ≥ h), the electrolyte droplets fill the micropillar gaps to form the Wenzel state. Then, the triphase interface disappears and McPCPFs cannot be obtained. Therefore, only superhydrophobic micropillar‐structured substrates with *f*_SL_ of 3.63–19.75% are suitable for constructing the McPCPFs. And the use of such substrates provides a very simple method to screen superhydrophobic substrates (>150°) suitable for the fabrication of McPCPFs through wettability characterization.

After electrochemical polymerization along the triphase interface, McPCPFs with the same geometrical parameters as the triphase interface were obtained (Figure S7, Supporting Information). The height of the microcavity was then measured using the corresponding profile of the McPCPF. As shown in Figure 2d, the height of the microcavity increased with an increase in the micropillar distance for micropillars with different diameters. For example, the height of the microcavities with base diameter of 3, 5, and 7 µm increases from 0.66 ± 0.15 µm for *D* = 4 µm to 2.5 ± 0.14 µm for *D* = 12 µm, from 1.43 ± 0.03 µm for *D* = 6 µm to 2.63 ± 0.14 µm for *D* = 12 µm, and from 2.05 ± 0.12 µm for *D* = 8 µm to 2.68 ± 0.55 µm for *D* = 12 µm. However, the micropillar diameter showed no obvious effect on the height of the microcavity, which may be due to their small differences.

To further demonstrate the diversity of the McPCPFs, electrochemical polymerization on superhydrophobic micropillar‐structured substrates with various geometrical parameters (e.g., shape and arrangement) was also carried out (Figure S8 and Table S4, Supporting Information). As shown in Figures S9, S10, and S11 (Supporting Information), 3D McPCPFs with different base shapes in different arrangements were fabricated using this strategy, including a circular base in a square arrangement with a diameter of 8 µm and different distances (i.e., 10, 15, and 20 µm), a hexagonal base in a hexagonal arrangement with a side length of 5 µm and different distances (i.e., 15, 20, and 25 µm), an anisotropic spindle base in a square arrangement with an *X*‐axis length of 10 µm, *Y*‐axis length of 7 µm, *X*‐axis distance of 13 µm, and different *Y*‐axis distances (i.e., 16, 22, and 27 µm), and a hierarchical circular base in a hexagonal arrangement with a diameter of 3 µm, different first‐level distances (i.e., 20 and 26 µm), and second‐level distances (i.e., 6 and 9 µm). Then, the corresponding profiles of these McPCPFs were used to measure the height of the microcavities (Figure S12, Supporting Information). With an increase in the micropillar distance, the height of the microcavities with a circular base in a square arrangement and a hexagonal base in a hexagonal arrangement will increase (Figure S13a,b, Supporting Information), which is similar to the trend of the height of microcavities with a circular base in a hexagonal arrangement. For the microcavities with a spindle base in a square arrangement, the height will increase from 4.62 ± 0.32 µm to 7.1 ± 0.61 µm in the *Y*‐axis direction and decrease from 3.58 ± 0.64 µm to 2.6 ± 0.23 µm in the *X*‐axis direction with the increase in the *Y*‐axis distance from 16 to 27 µm, showing an evident anisotropy (Figure S13c, Supporting Information). Besides, the McPCPFs constructed on superhydrophobic clusters of circular micropillars in a hexagonal arrangement exhibited distinct hierarchical structures (Table S4, Supporting Information). Taking hierarchical microcavities on substrate i for example, the corresponding first‐level height and second‐level height are 5.93 ± 0.28 µm and 0.84 ± 0.16 µm, respectively. Therefore, the existence of the solid/liquid/gas triphase interface on the superhydrophobic micropillar‐structured substrates facilitates successful construction of McPCPFs, and the geometrical parameters of the McPCPFs can be further mediated by changing the geometrical parameters of the substrates.

### 2.3 Epitaxial Growth along the Triphase Interface

**Figure 3:**
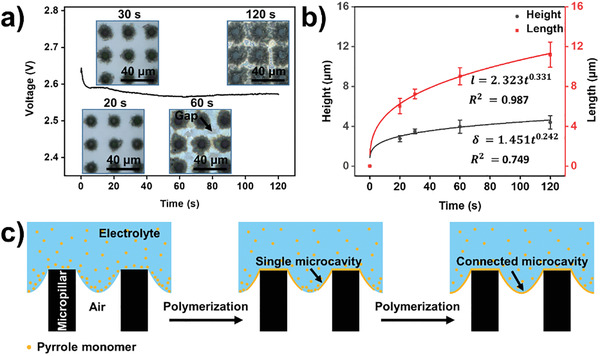
Epitaxial growth of the McPCPFs along the triphase interface. a) The voltage versus polymerization time curve of McPCPFs. The inset images are the corresponding plane laser‐scanning microscopic images at different polymerization times. b) The height and projected length of the microcavities increase with the increase in the polymerization time. The dependence of the height and projected length of the microcavities on the polymerization time follow the equations of *δ* = 1.451*t* ^0.242^ and *l* = 2.323*t* ^0.331^, where *δ*, *l*, and *t* are the height, projected length and the polymerization time, respectively. c) Schematic illustration of the epitaxial growth of the McPCPFs along the triphase interface. The pyrrole monomers with hydrophobicity tend to aggregate at the triphase interface (left). The single 3D microcavities show evident epitaxial growth along the triphase interface (middle). With the increase in polymerization time, they become connected to form the intact McPCPFs (right).

To investigate the McPCPF growth mechanism on superhydrophobic micropillar‐structured substrates, the process of electrochemical polymerization of the McPCPFs with a circular base in a square arrangement on micropillar‐structured substrates with a diameter of 8 µm and a distance of 20 µm was studied. As shown in **Figure** **3**a, the 3D microcavities grew gradually with increasing polymerization time. For polymerization times of 20 and 30 s, single unconnected microcavities formed on the substrate. With an increase in the polymerization time, microcavity growth continued until they connected with each other. At a polymerization time of 60 s, gaps still remained in the McPCPFs. When the polymerization time reached 120 s, the gaps disappeared, indicating the successful formation of intact McPCPFs. The height and projected length of these microcavities were measured using their profiles (Figure S14, Supporting Information). They increased from 2.76 ± 0.31 and 6.03 ± 0.77 µm at 20 s to 4.39 ± 0.67 and 11.19 ± 1.27 µm at 120 s, evidently exhibiting epitaxial growth along the triphase interface (Figure 3b). The preferential epitaxial growth process is illustrated in Figure 3c. After the introduction of the HDBS surfactant (Figure S15, Supporting Information), the lower surface tension of the electrolyte droplet causes the formation of a triphase interface with a meniscus height *δ* on the micropillar‐structured substrate. Furthermore, the hydrophobic pyrrole monomers tended to aggregate at the triphase interface (left in Figure 3c).^[^ ^28^ ^]^ Electrochemical oxidation polymerization occurs at the triphase interface when the experimental setup is powered on, while electrochemical reduction hydrogen evolution occurs at the platinum ring electrode. The corresponding chemical equations are as follows:

 
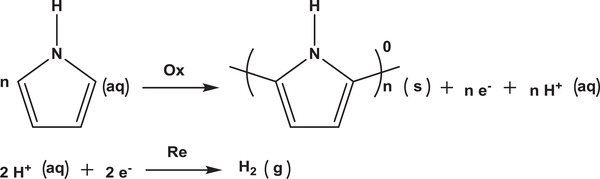


Specifically, the pyrrole monomers at the interface are first oxidized to cationic radicals. Subsequently, the cationic radicals couple to form dimers and polymers, and further form single 3D microcavities, exhibiting epitaxial growth along the triphase interface (middle in Figure 3c).^[^ ^29^ ^]^ Finally, the single 3D microcavities connect to form intact McPCPFs with the increase in polymerization time (right in Figure 3c). To ensure the integrity of the free‐standing 3D McPCPFs after peeling and transfer, the polymerization times were generally maintained at 300 s to obtain McPCPFs with a thickness of 0.151 ± 0.017 µm (Figures S16 and S17, Supporting Information). Therefore, the preferential epitaxial growth along the solid/liquid/gas triphase interface leads to the formation of the McPCPFs.

The effects of other surfactant electrolytes and conducting polymer monomers on the construction of McPCPFs were also investigated to test the universality of this strategy. While maintaining the same surfactant concentration, electrolyte droplets containing alternative surfactants, such as sodium dodecyl sulfate (SDS) and hexadecyl trimethyl ammonium bromide (CTAB) were employed for fabricating patterned PPy under the same constant current. Only, partially connected PPy microcavities and individual PPy patterns solely at the top of the micropillars were obtained, because the penetration and filling of the electrolyte droplets during the electrochemical polymerization process inhibited further growth (Figure S18, Supporting Information). Similar cases occurred when surfactant electrolyte droplets containing 0.01 m HDBS and other conducting polymer monomers, such as 3,4‐ethylenedioxythiophene and aniline were used. Partially connected poly(3,4‐ethylenedioxythiophene) (PEDOT) microcavities and individual polyaniline (PANI) patterns formed only at the top of the micropillars (Figure S19, Supporting Information). However, the corresponding heterogeneous films were successfully fabricated through a two‐step electrochemical reaction (Figure S20, Supporting Information). For example, a second electrochemical reaction on the as‐prepared PPy film was employed to obtain the organic/organic (i.e., PPy/PEDOT) and organic/inorganic (i.e., PPy/CuS) heterogeneous films with microcavities (Figure S21, Supporting Information). Theoretically, if there is a stable triphase interface on the superhydrophobic substrates during the electrochemical reaction process, then this strategy can be employed to construct patterned films. However, intermediate products (e.g., dimers and polymers) or gas products are usually produced during this process, which affects the stability of the interface. If the wettability of the electrolyte droplets on the substrates changes from the Cassie state to the Wenzel state in a short time, only single patterns are formed. When the interface is stable for a sufficiently long time, patterned films are preferred. Thus this is a general patterning strategy to some degree and is not specifically limited to conducting polymers.

### 2.4 Easy‐Peeling and Nondestructive Transfer Properties

**Figure 4:**
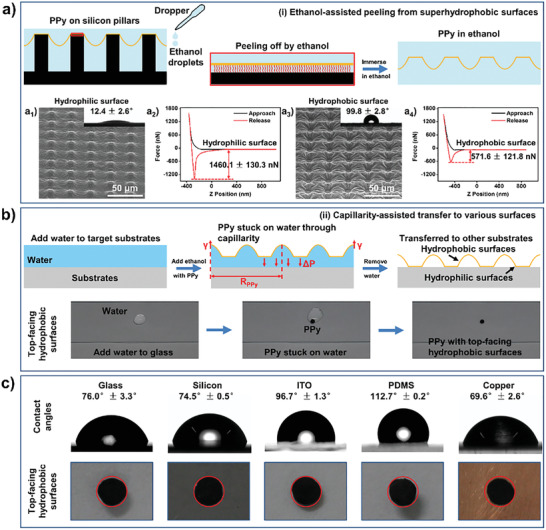
Ethanol‐assisted peeling from superhydrophobic surfaces and capillarity‐assisted nondestructive transfer to various substrates. a) The peeling off process from superhydrophobic surfaces through adding ethanol droplets (top). Environmental scanning electron microscope (ESEM) images of the hydrophilic surface a_1_) and the hydrophobic surface a_3_) of the McPCPFs. The inset optical images show the corresponding water droplet contact angles, respectively. The typical force curves of the hydrophilic surface a_2_) and the hydrophobic surface a_4_). The red wavy lines represent FAS molecules modified on the substrate. b) The nondestructive transfer process of the peeled off McPCPFs with a macroscopic diameter of 2.27 ± 0.19 mm (Figure S23, Supporting Information) under the assistance of capillarity, which includes adding a water droplet to the targeted substrate, adding an ethanol droplet with the McPCPF and removing the water on the substrate in sequence (top). *γ*, *R_PPy_*, and Δ*P* represent the surface tension of water, the macroscopic radius of the whole McPCPF and the Laplace pressure, respectively. The transfer process of the McPCPF with the top‐facing hydrophobic surface to the glass (bottom). c) The McPCPFs nondestructively transferred to substrates with different wettability. The optical images of the water droplet contact angles (top) of these substrates and the McPCPFs with the top‐facing hydrophobic surface (bottom) on the corresponding substrates.

The as‐prepared McPCPFs exhibited easy‐peeling property, whereby the addition of ethanol droplets on the superhydrophobic micropillar‐structured substrates facilitated separation of the films (top in **Figure** **4**a). This may be caused by the weakening of the adhesion between the McPCPFs and the superhydrophobic substrates due to the insertion of ethanol.^[^ ^30^ ^]^ In addition, the two McPCPF surfaces showed asymmetric wettability. Taking the McPCPFs with a circular base in a square arrangement in base diameter of 8 µm and distance of 20 µm for example, the surface inside the electrolyte droplet (i.e., the hydrophilic surface) and the surface outside the electrolyte droplet (i.e., the hydrophobic surface) exhibit water droplet contact angles of 12.4 ± 2.6° and 99.8 ± 2.8°, respectively (Figure 4a_1_,a_3_). This asymmetric wettability was further investigated via a measurement of the adhesion force of the two surfaces using an atomic force microscope probe with hydroxyl groups (Figure 4a_2_,a_4_), showing a higher adhesion force of 1460.1 ± 130.3 nN and a lower adhesion force of 571.6 ± 121.8 nN on the hydrophilic and hydrophobic surfaces, respectively. This is attributed to there being more hydrophilic benzene sulfonic acid functional groups of the DBS^−^ anions on the hydrophilic surface as evidenced by the greater S 2p signal (Figure S22, Supporting Information), which can generate stronger adhesion with the probe decorated with hydroxyl groups. This type of Janus wettability can contribute to the subsequent nondestructive transfer to different substrates.

After the process of ethanol‐assisted peeling from superhydrophobic surfaces, the capillarity‐assisted nondestructive transfer to various substrates can be achieved (top of Figure 4b). In detail, a ≈50 µL water droplet was added to the targeted substrate, followed by the addition of an ethanol droplet containing the McPCPF using a dropper. The McPCPF is then generally stuck on the surface of the water through capillarity (*F*) on the hydrophilic surface.^[^ ^31^ ^]^ After slowly removing the water on the substrate using a pipette, the McPCPF will be attached to the substrate with a top‐facing hydrophobic surface. Taking the glass substrate for example, the McPCPF with a top‐facing hydrophobic surface was transferred to the glass surface using the abovementioned method (bottom of Figure 4b). Besides, the McPCPF could also be transferred to the glass surface with a top‐facing hydrophilic surface through moving the glass substrate onto the hydrophobic surface of the McPCPF on the surface of the water and subsequently moving the glass substrate upward (Figure S24a, Supporting Information). Furthermore, nondestructive transfer of the McPCPFs with both top‐facing hydrophobic and hydrophilic surfaces to various substrates with different wettability was also demonstrated, which include inorganic (i.e., glass, silicon, indium tin oxide (ITO)), organic (i.e., PDMS), and metal (i.e., copper) substrates (Figure 4c; and Figure S24b, Supporting Information). Although these films underwent partial wrinkling after the transfer process, the microcavities did not change (Figure S25, Supporting Information). However, these films were not robust because they were very thin and they could not withstand tearing. As shown in Figure S26 (Supporting Information), they could only withstand slight deformation following transfer to the viscoelastic PDMS via imprinting, owing to the microcavity structure. With an increase in the polymerization time, thicker McPCPFs with better mechanical property were obtained, which could withstand repeated folding and spreading during the process of entering into and out of the water (Figure S27, Supporting Information). Thus, the McPCPFs can be readily transferred to various substrates with different wettability through ethanol‐assisted peeling from superhydrophobic surfaces and capillarity‐assisted nondestructive transfer.

Electrochemical polymerization along the triphase interface as a strategy to construct patterned films, the challenges in preparing larger McPCPFs are also considered. As shown in Figure S23 (Supporting Information), the whole McPCPFs showed an almost circular shape with a macroscopic diameter of 2.27 ± 0.19 mm, which is the largest film obtained after polymerization for 300 s using 20 µL electrolyte droplets. In theory, larger McPCPFs can be obtained through increasing the contact area between the electrolyte droplets and the superhydrophobic substrates with an increase in the droplet volume. However, it is difficult to maintain a stable triphase interface, especially for surfactant electrolyte droplets. Because that these droplets with low surface tension tended to penetrate and fill the micropillar gaps under the action of hydrostatic pressure or the stimulation of external vibration. Although, it may be possible to enhance the production of these films by repeating the polymerization process in a printing manner or by combining droplet arrays on superhydrophobic substrates and electrochemical polymerization.

### 2.5 Demonstration of McPCPFs in Light‐Driven Locomotion

**Figure 5:**
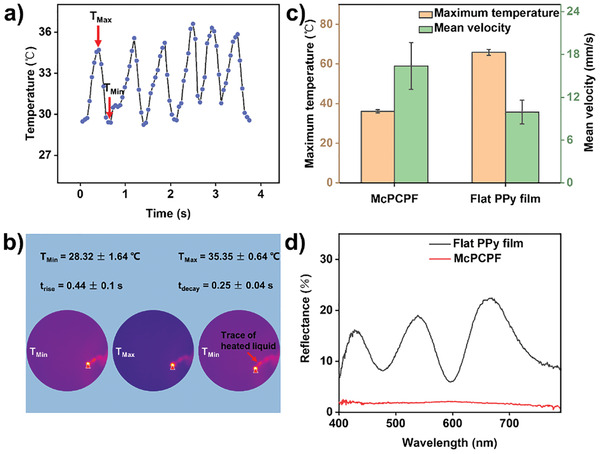
Demonstration of McPCPFs in light‐driven locomotion on organic liquid surfaces. a) The temperature versus time curve of McPCPFs during six repeated heating/cooling cycles on DMSO surfaces. When the McPCPFs were heated from the minimum temperature *T*_Min_ to the maximum temperature *T*_Max_, they begin to locomote. When the temperature decreases to *T*_Min_, the McPCPFs gradually stop locomoting. b) The typical infrared thermal images of the McPCPFs at *T_Min_* and T*_Max_* during one repeated heating/cooling cycle. The corresponding time differences from T*_Min_* to T*_Max_* and from T*_Max_* to T*_Min_* are recorded as t*_rise_* and t*_decay_*, respectively. The red triangle and arrow represent the position of the McPCPF and the trace of heated liquid, respectively. c) Maximum temperature T*_Max_* and the corresponding mean velocity when the films locomote. d) Reflectance spectra of the flat films and McPCPFs.

Inspired by water striders that can move freely on water surfaces, we demonstrated light‐driven locomotion on organic liquid (i.e., dimethyl sulfoxide (DMSO)) surfaces using McPCPFs based on photothermal conversion. Under 532 nm laser irradiation with a power density of ≈0.9 W cm^−2^, the McPCPFs on liquid surfaces will be heated to increase their temperature due to photothermal conversion. The generated heat can be delivered to the contact interface between the liquid and the McPCPF, resulting in an increase in the temperature of the liquid and the generation of hot vapors.^[^ ^32^ ^]^ When the hot vapors can produce sufficient driving force to overcome the resistance on the liquid surface, the McPCPF will locomote. As shown in **Figure** **5**a,b, when the McPCPF with a circular base in a square arrangement in diameter of 8 µm and distance of 10 µm were heated from the minimum temperature *T*_Min_ to the maximum temperature *T*_Max_, enough hot vapors were generated to promote their locomotion on the DMSO surface. When the temperature decrease to *T*_Min_, less hot vapors were generated and the McPCPF locomotion gradually stopped. The results indicated that the light‐driven locomotion of the McPCPFs showed a lower *T*_Max_ of 35.35 ± 0.64 ℃, lower *t*_rise_ of 0.44 ± 0.1 s and *t*_decay_ of 0.25 ± 0.04 s, and a higher mean velocity of 13.48 ± 2.28 mm s^−1^ (Figure 5c). As a comparison, the locomotion of the flat conducting polymer films constructed on flat silicon substrates exhibited a higher *T*_Max_ at 65.86 ± 1.51 ℃, higher *t*_rise_ of 1.46 ± 0.49 s and *t*_decay_ of 0.56 ± 0.1 s, as well as a lower mean velocity of 9.93 ± 1.67 mm s^−1^ (Figure S28, Supporting Information). This is because the McPCPFs can more effectively convert light to heat due to their lower reflectance taking advantage of the microcavity structure in contrast to the flat films with low surface roughness of ≈0.018 µm (Figure 5d; and Figure S29, Supporting Information).^[^ ^2a^ ^]^ Thus, these McPCPFs can generate enough hot vapors to overcome the resistance on DMSO surfaces at lower temperatures. Therefore, in contrast to the flat counterparts, the as‐prepared McPCPFs show more sensitive light‐driven locomotion on organic liquid surfaces through effective photothermal conversion. What's more, these films can also be employed in other fields, such as detecting ammonia gas (Figure S30, Supporting Information).

## 3 Conclusion

In conclusion, we proposed a strategy for the superhydrophobic‐substrate‐assisted construction of free‐standing 3D McPCPFs at micrometer resolution with easy‐peeling and nondestructive transfer properties. First, McPCPFs were constructed on the superhydrophobic micropillar‐structured substrates with various geometrical parameters (e.g., diameter, distance, shape, and arrangement), suggesting that superhydrophobic substrates (>150°) with *f*_SL_ of 3.63–19.75% are suitable for constructing these films. Then, the epitaxial growth along the solid/liquid/gas triphase interface at different electrochemical polymerization times was conducted and the universality of this strategy was discussed. Besides, ethanol‐assisted peeling from superhydrophobic surfaces and capillarity‐assisted transfer of the McPCPFs to various substrates, as well as the challenges in preparing larger films were provided. Furthermore, sensitive light‐driven locomotion of the as‐prepared McPCPFs in contrast to the flat counterparts on organic liquid surfaces was demonstrated. Overall, a facile strategy for the construction of free‐standing 3D microstructure‐patterned conducting polymer films has been developed, which will improve productivity and promote application in the fields of actuation, water purification, sensing, and bioelectronics.

## 4 Experimental Section

### Silanization Treatment of Micropillar‐Structured Substrates

N‐type silicon wafers (10 cm diameter, <100> oriented, P doped, 0.001–0.003 Ω cm resistivity) were employed to construct micropillars with different shapes in different arrangements through a direct laser writing apparatus (Heidelberg Instruments DWL200, Germany), which include a square arrangement of circular micropillars (diameter of 8 µm, height of 20 µm, and distances of 10, 15, and 20 µm), a hexagonal arrangement of circular micropillars (diameters of 3, 5, and 7 µm, height of 10 µm and distances of 2, 4, 6, 8, 10, and 12 µm), a hexagonal arrangement of hexagonal micropillars (side length of 5 µm, height of 15 µm, and distances of 15, 20, and 25 µm), a square arrangement of spindle micropillars (*X*‐axis length of 10 µm and *Y*‐axis length of 7 µm, height of 20 µm, *X*‐axis distance of 13 µm, and *Y*‐axis distances of 16, 22, and 27 µm), and a hexagonal arrangement of circular micropillar clusters (diameter of 3 µm, height of 15 µm, first‐level distances of 20 and 26 µm, and second‐level distances of 6 and 9 µm). To ensure the conductivity of the micropillar‐structured substrates, the silicon wafers were immersed into a 1% HF solution for 10 min to remove the silica layer on the surfaces. Then, these micropillar‐structured substrates were modified with a monolayer of heptadecafluoro‐decyltrimethoxysilane (FAS) molecules in a decompression environment at 80 °C for 12 h.

### Superhydrophobic‐Substrate‐Assisted Construction of McPCPFs

The experimental setup for constructing 3D McPCPFs consists of a platinum ring as a counter electrode, an electrolyte droplet containing conducting polymer monomers and electrolyte, a superhydrophobic micropillar‐structured substrate as a working electrode, as well as a Keithley 2611B power source. In detail, a platinum ring with a macroscopic diameter of ≈3.7 mm was first placed above the micropillar‐structured substrate with a distance of ≈1.6 mm. Then, a 20 µL electrolyte droplet containing 0.24 m pyrrole and 0.01 m dodecylbenzene sulfonic acid (HDBS) was slowly added to the platinum ring using a pipette, forming a connection with the superhydrophobic micropillar‐structured substrate below. After that the experimental setup was powered with a constant current of 20 µA and the 3D McPCPFs were polymerized on the micropillar‐structured substrates. Electrolyte droplets (20 µL) consisting of 0.24 m pyrrole and 0.01 m sodium dodecyl sulfate (SDS), and 0.24 m pyrrole and 0.01 m hexadecyl trimethyl ammonium bromide (CTAB) were also employed for constructing patterned polypyrrole (i.e., PPy) with the same constant current. Besides, electrolyte droplets (20 µL) containing 0.01 m 3,4‐ethylenedioxythiophene (i.e., EDOT) and 0.01 m HDBS, and 0.2 m aniline, and 0.01 m HDBS were used for constructing patterned poly(3,4‐ethylenedioxythiophene) (i.e., PEDOT) and polyaniline (i.e., PANI) with the same constant current, respectively. What's more, the corresponding heterogeneous films were also successfully fabricated via a two‐step electrochemical reaction. In detail, a 20 µL electrolyte droplet consisting of 0.01 m EDOT and 0.1 m HClO_4_ was dropped on an as‐prepared PPy film. After electropolymerization with a current of 20 µA for 600 s, an organic/organic heterogeneous film (i.e., PPy/PEDOT) with microcavities was obtained. Alternatively, a 250 *μ*L aqueous solution with 0.1 m HCl was added to a 2.5 mL aqueous solution with 0.02 m CuCl_2_, 0.02 m Na_2_S_2_O_3_, and 0.02 m C_6_H_5_Na_3_O_7_ to form the mixed electrolyte solution. Then, a 20 µL electrolyte droplet was dropped on the PPy film for the second electrochemical deposition at a current of 0.1 mA for 600 s, resulting in the preparation of an organic/inorganic heterogeneous film (i.e., PPy/CuS) with microcavities.

### Characterization

The 3D and plane morphologies of the McPCPFs and the corresponding profiles were characterized using a nanosearch microscope (Olympus OLS‐4500, Japan). The environmental scanning electron microscope (ESEM) images of the 3D McPCPFs were obtained by an environmental scanning electron microscopy (FEI 10 Quanta 200, America). The typical force curves of the McPCPFs were obtained using an atomic force microscope (Shimadzu SPM‐9700, Japan) under contact mode. An atomic force microscope probe (BudgetSensors Tap300Al‐G, Bulgaria) with hydroxyl groups was employed, and the corresponding scan rate was 2 Hz and the scan size was 2 × 2 µm^2^. The reflectance spectra of the conducting polymer films were measured through an Ocean Optics Maya 2000 PRO fiber optic spectrometer. X‐ray photoelectron spectroscopy was obtained using a Thermo Scientific ESCALab 250Xi (ESCALab250Xi, Thermo Scientific, America). The light‐driven locomotion on organic liquid surfaces of the as‐prepared McPCPFs was performed using a laser (NBT‐532) with the power density of ≈0.9 W cm^−2^. The infrared thermal images were obtained using an infrared thermal imaging camera (FLIR A615). A 10 nm layer of Cr and a 100 nm layer of Au were sequentially deposited on the McPCPF with the assistance of the copper mesh mask to form the NH_3_ detector using an e‐beam evaporator (Ohmiker‐50B, Cello Technology, Taiwan). The voltage versus current curve of the NH_3_ detector without NH_3_ was obtained using a Keithley 4200‐SCS in the range of −0.1 and 0.1 V, and the real‐time resistance change was recorded under a constant potential of 0.1 V after applying 300 ppm NH_3_. The wettability of the micropillar‐structured substrates and the McPCPFs were investigated through an OCA 25 machine (Dataphysics OCA25, Germany) at room temperature in air with water droplets of ≈3 and 0.2 µL, respectively. The surface tension of water and the electrolyte solution with 0.01 m HDBS was explored by the OCA 25 machine. Specifically, the droplets were squeezed out from a needle in diameter of ≈1.6 mm and the shapes of the pendant droplets were captured and calculated for surface tension as the volumes of the droplet reached the maximum value.

## Conflict of Interest

The authors declare no conflict of interest.

## Supporting information

Supporting Information

## Data Availability

Research data are not shared.
