# Supplementary material for: Superhydrophobic‐Substrate‐Assisted Construction of Free‐Standing Microcavity‐Patterned Conducting Polymer Films
Source: Adv Sci (Weinh). 2021 Jul 10;8(17):2100949. doi: 10.1002/advs.202100949 (PMC8425917; doi:10.1002/advs.202100949)
Supplement: Supplementary file 1 — Supporting Information [file ADVS-8-2100949-s001.pdf]

WILEY-VCH

© 2021 Wiley-VCH GmbH

ADVANCED SCIENCE
Open Access

Supporting Information

for *Adv. Sci.,* DOI: 10.1002/advs.202100949

Superhydrophobic-Substrate-Assisted Construction of Free-Standing Microcavity-Patterned Conducting Polymer Films

Yupeng Chen, Zhongpeng Zhu, Xiangyu Jiang and Lei Jiang**

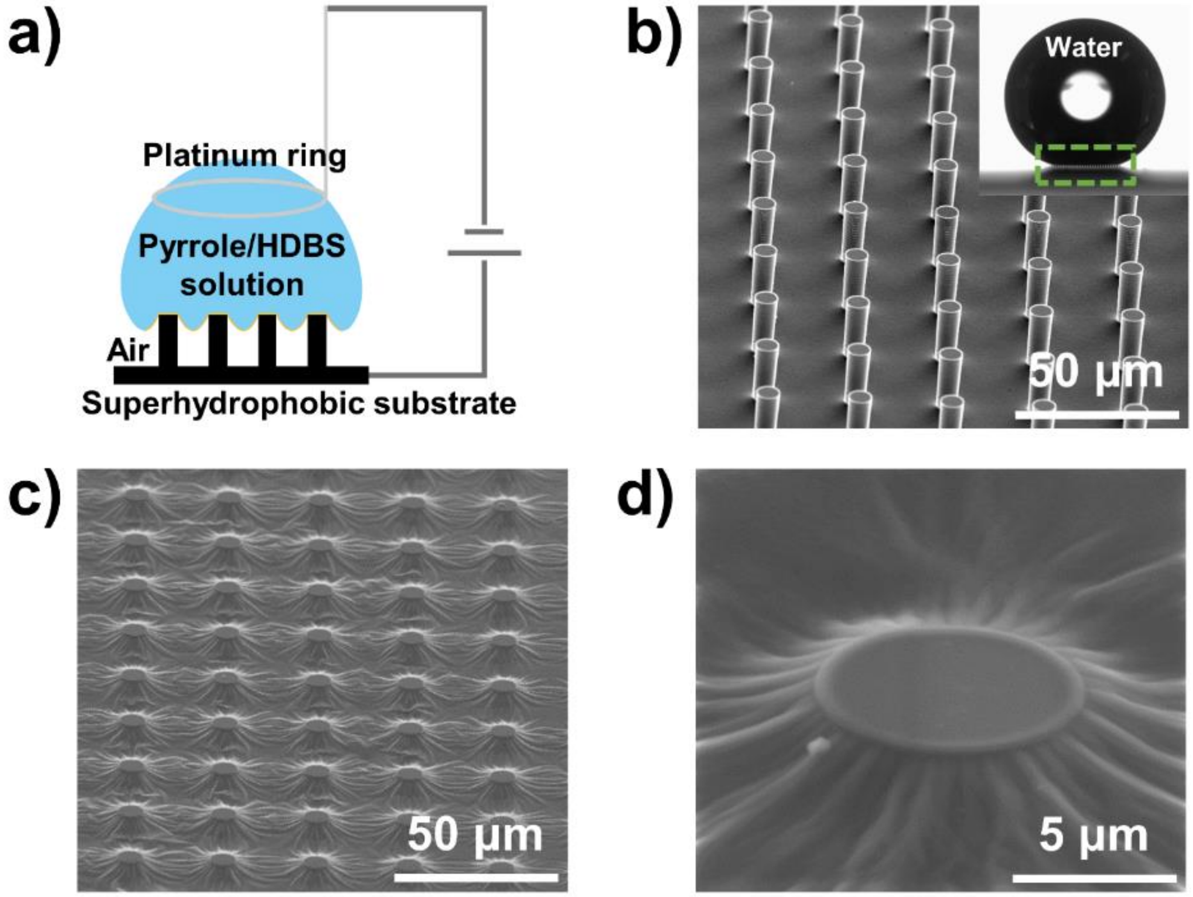

Figure S1. McPCPFs with a circular base in a square arrangement polymerized on micropillar-structured substrates with a square arrangement of circular micropillars. a) Schematic illustration of the construction of McPCPFs along the solid/liquid/gas triphase interface through the experimental setup. b) ESEM image of superhydrophobic micropillar-structured substrates with a square arrangement of circular micropillars. The inset optical image shows the superhydrophobicity of the micropillar-structured substrates with a contact angle of 154.8 ± 0.6°. The green box shows the triphase interface consisting of the micropillars, air and the water droplet. c) ESEM image of McPCPFs (i.e., PPy) with a circular base in a square arrangement polymerized on the micropillar-structured substrates. d) Enlarged ESEM image of an individual microcavity.

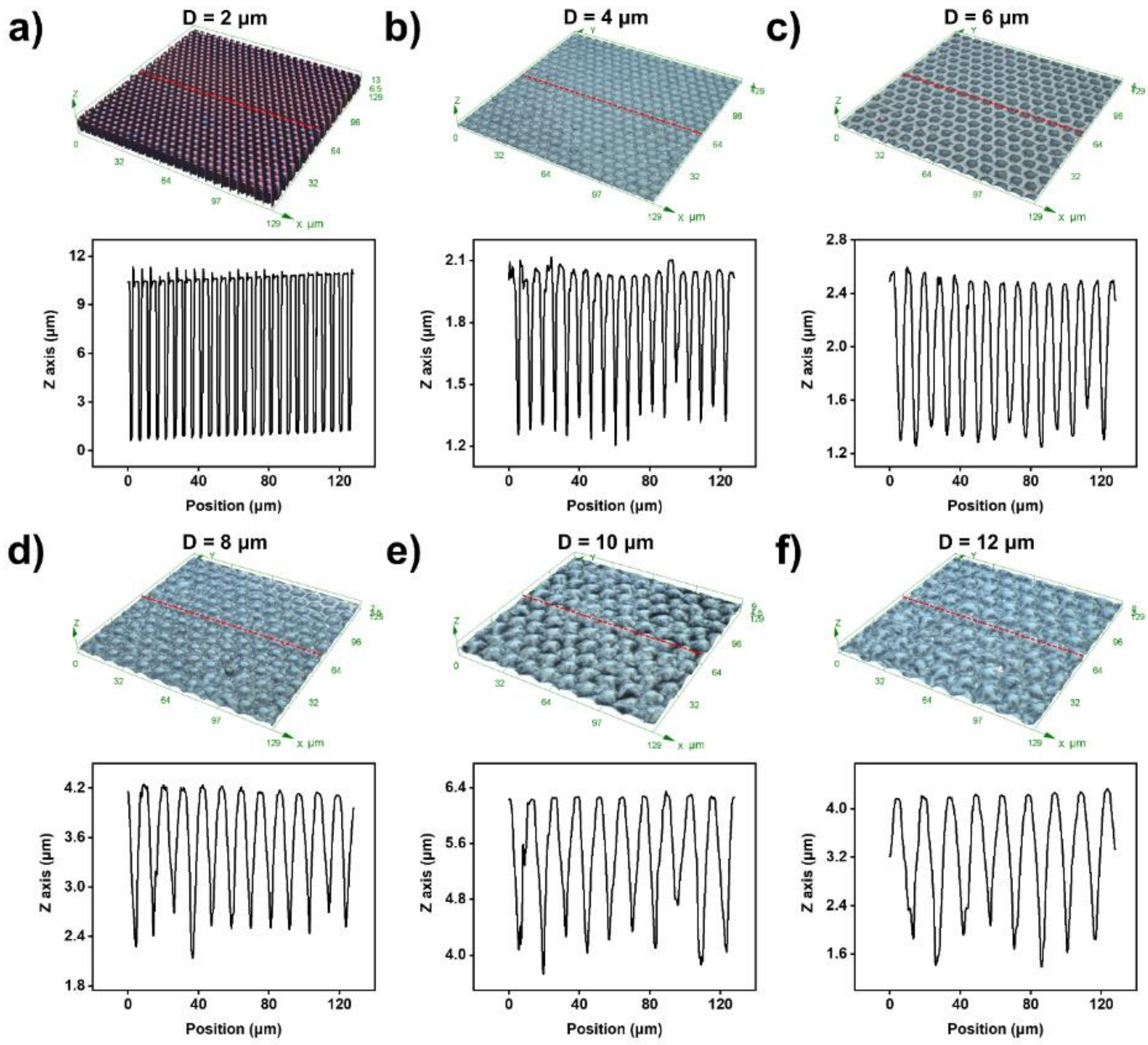

Figure S2. The dependence of McPCPFs on the micropillar distance of substrates with a hexagonal arrangement of circular micropillars in diameter of 3 μm. 3D laser-scanning microscopic images and the corresponding profiles along the red dotted lines after electrochemical polymerization on the micropillar-structured substrates with distances of 2 a), 4 b), 6 c), 8 d), 10 e) and 12 μm f). For the micropillar-structured substrates with distance of 2 μm a), the McPCPFs were not formed.

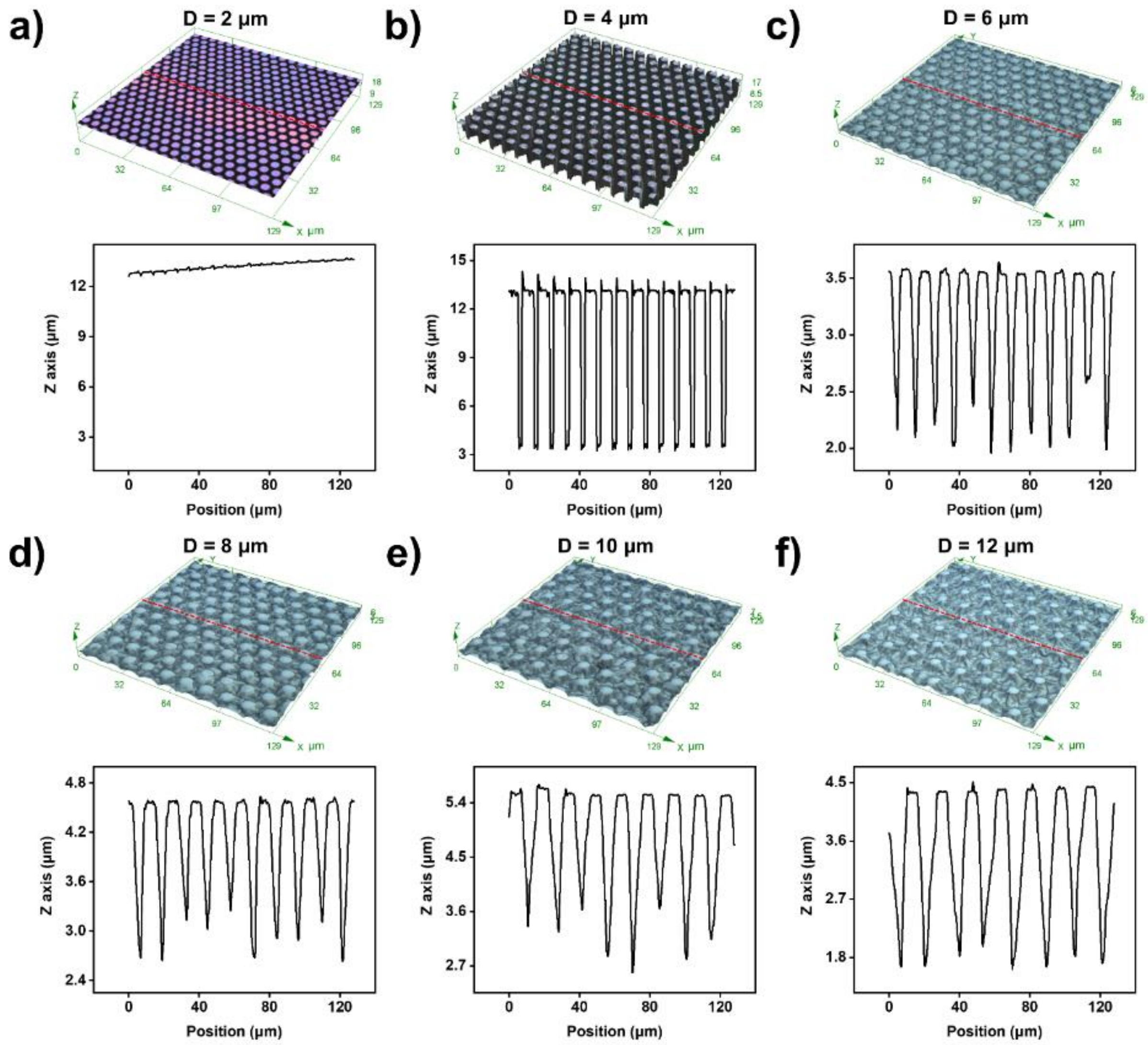

Figure S3. The dependence of McPCPFs on the micropillar distance of substrates with a hexagonal arrangement of circular micropillars in diameter of 5 µm. 3D laser-scanning microscopic images and the corresponding profiles along the red dotted lines after electrochemical polymerization on the micropillar-structured substrates with distances of 2 a), 4 b), 6 c), 8 d), 10 e) and 12 µm f). For the micropillar-structured substrates with distances of 2 a) and 4 µm b), the McPCPFs were not formed.

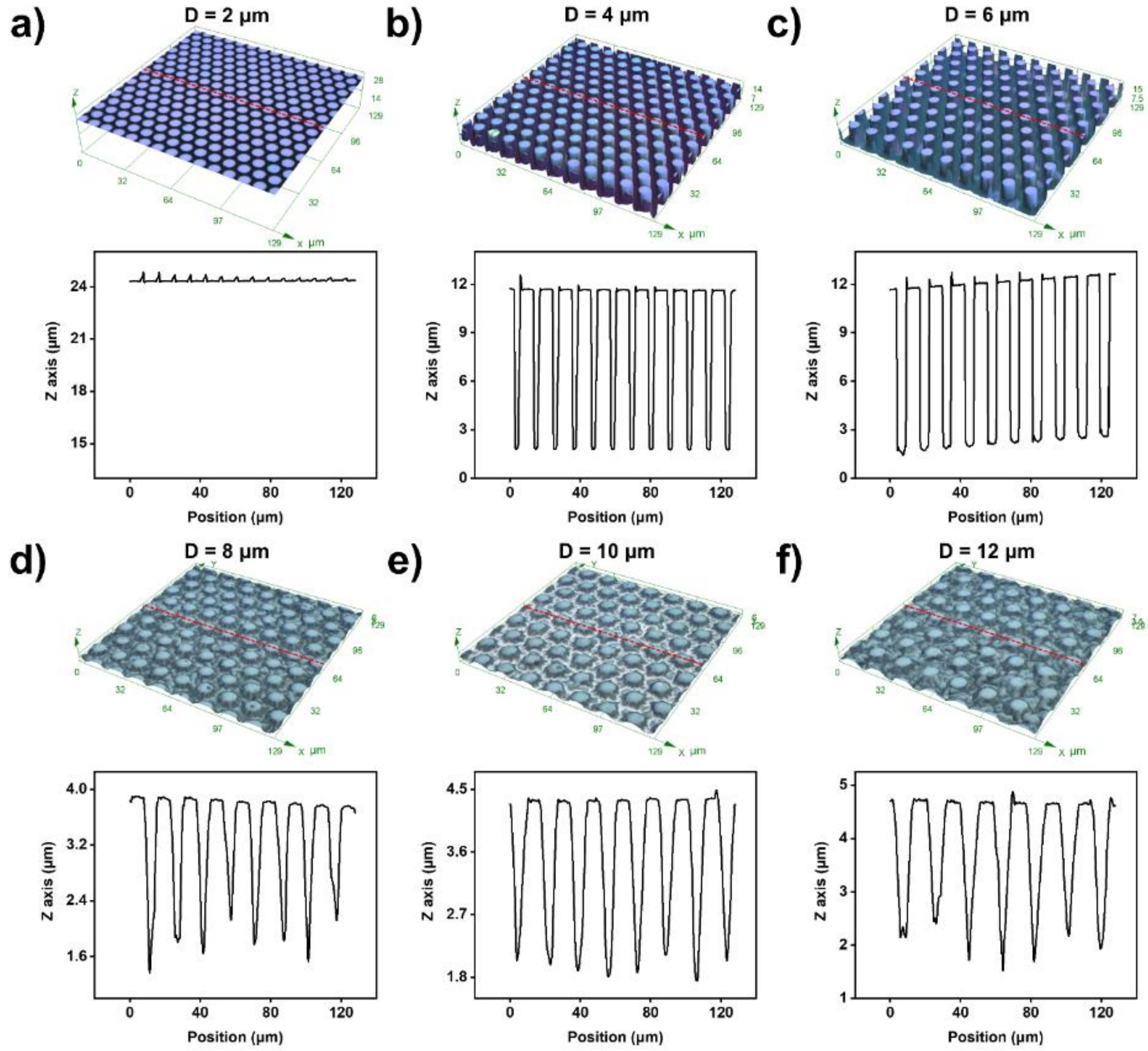

Figure S4. The dependence of McPCPFs on the micropillar distance of substrates with a hexagonal arrangement of circular micropillars in diameter of 7 μm. 3D laser-scanning microscopic images and the corresponding profiles along the red dotted lines after electrochemical polymerization on the micropillar-structured substrates with distances of 2 a), 4 b), 6 c), 8 d), 10 e) and 12 μm f). For the micropillar-structured substrates with distances of 2 a), 4 b) and 6 μm c), the McPCPFs were not formed.

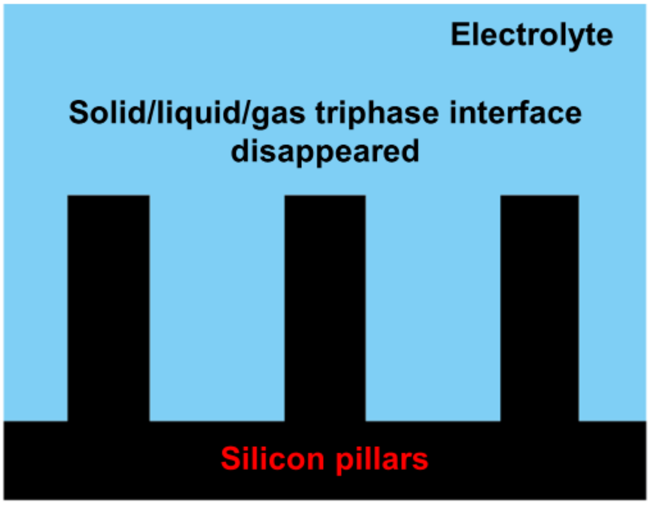

Figure S5. Schematic illustration of the Wenzel state when the surfactant electrolyte droplets filled the micropillar gaps and the solid/liquid/gas triphase interface disappeared.

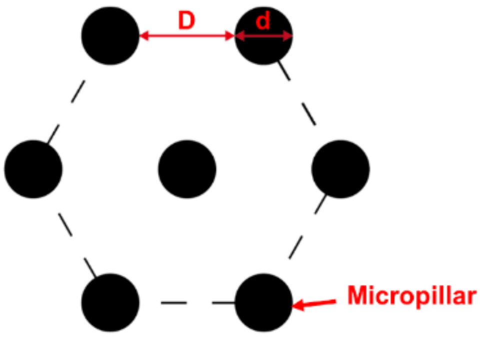

Figure S6. Schematic illustration of micropillar-structured substrates with a hexagonal arrangement of circular micropillars, where *D* and *d* represent the micropillar distance and diameter, respectively.

According to the critical angle theory proposed by Quere *et al.* (J. Bico, U. Thiele, D. Quere, Colloid. Surface. A. 2002, 206, 41; A. Lafuma, D. Quere, Nat. Mater. 2003, 2, 457.), critical angles for micropillar-structured substrates can be calculated by the following equation:

$$\cos\theta_c = (f_{SL} - 1)/(r - f_{SL}) \quad (1)$$

where the f_{SL} is the fractional flat geometrical area of the solid/liquid interface under a droplet, and r is the surface roughness of the micropillar-structured substrates, θ_c is the critical contact angles for these substrates, representing the threshold value between the Cassie state and the Wenzel state. As shown in Table 3, the calculated critical angles of these substrates were <150°. The corresponding measured angles were larger than these calculated angles. According to the critical angle theory proposed by Quere *et al.*, the droplets on these substrates should be at the Cassie state and there will be no penetration. However, only the substrates with larger measured angles (>150°) can be used to construct McPCPFs in the practical experiment. Notably, the surfactant electrolyte droplets with the low surface tension were employed for constructing McPCPFs on these substrates. Thus, larger angles were needed to ensure the stable existence of the triphase interface during the process of

electropolymerization, because that surfactant electrolyte droplets tended to penetrate and fill the micropillar gaps under the action of hydrostatic pressure or the stimulation of external vibration. Even though, the critical angle theory can still provide a guidance for the pre-design of the micropillar-structured substrates which were favorable to construct McPCPFs.

The critical angles of different substrates were computed as follows:

For substrates with a hexagonal arrangement of circular micropillars (h = 10 μm) (Figure S6), the f_{SL} and r can be obtained through the following equations (2) and (3), where D, d and h are the micropillar distance, diameter and height, respectively. The results are as shown in Table 1 and Table 2.

$$f_{SL} = \pi d^2/2\sqrt{3}(D+d)^2 \quad (2)$$

$$r = 1 + 2\pi dh/\sqrt{3}(D+d)^2 \quad (3)$$

Then, we calculated the critical contact angles (θ_c) for these micropillar-structured substrates through the equation (1). The corresponding results are shown in Table 3, exhibiting lower critical contact angles compared to the measured angles in the experiment.

Table 1. Fractional flat geometrical area of the solid/liquid interface (f_{SL}) of substrates with a hexagonal arrangement of circular micropillars.

Distance (µm) / Diameter (µm)	2	4	6	8	10	12
**3**	**32.65%**	**16.66%**	**10.08%**	**6.75%**	**4.83%**	**3.63%**
**5**	**46.27%**	**27.99%**	**18.74%**	**13.42%**	**10.08%**	**7.85%**
**7**	**54.86%**	**36.73%**	**26.29%**	**19.75%**	**15.38%**	**12.31%**

Table 2. Surface roughness (*r*) of substrates with a hexagonal arrangement of circular micropillars.

Distance (µm) / Diameter (µm)	2	4	6	8	10	12
3	5.35	3.22	2.34	1.90	1.64	1.48
5	4.70	3.24	2.50	2.07	1.81	1.63
7	4.13	3.10	2.50	2.13	1.88	1.70

Table 3. Critical contact angles (θ_c) of substrates with a hexagonal arrangement of circular micropillars.

Diameter (µm) \ Distance (µm)	2	4	6	8	10	12
**3**	**97.7°**	**105.8°**	**113.6°**	**120.6°**	**126.6°**	**131.7°**
**5**	**97.3°**	**104.1°**	**110.6°**	**116.5°**	**121.8°**	**126.5°**
**7**	**97.2°**	**103.4°**	**109.2°**	**114.6°**	**119.4°**	**123.7°**

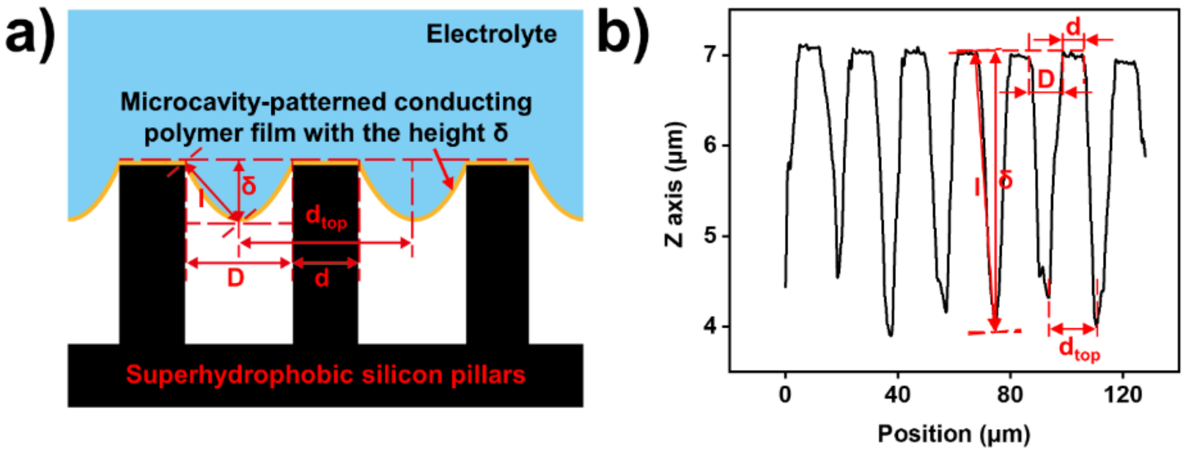

Figure S7. Geometrical parameters of McPCPFs can be obtained directly from the corresponding profiles. a) Schematic illustration of the McPCPFs polymerized along the solid/liquid/gas triphase interface consisting of the micropillars, air and the electrolyte droplet. The height, distance and base diameter of the microcavities are δ, D and d, which are determined by the meniscus height, the micropillar distance and diameter, respectively. The top diameter and the projected length of the microcavity are represented by d_{top} (the value is equal to the sum of the micropillar distance and diameter) and l (the value is fixed at the specific meniscus height and the micropillar distance), respectively. b) The typical profile of the McPCPF constructed on the micropillar-structured substrate with a circular base in a hexagonal arrangement (diameter of 7 μm and distance of 12 μm) for obtaining the corresponding geometrical parameters such as the height, distance, base diameter, top diameter and the projected length of the microcavities.

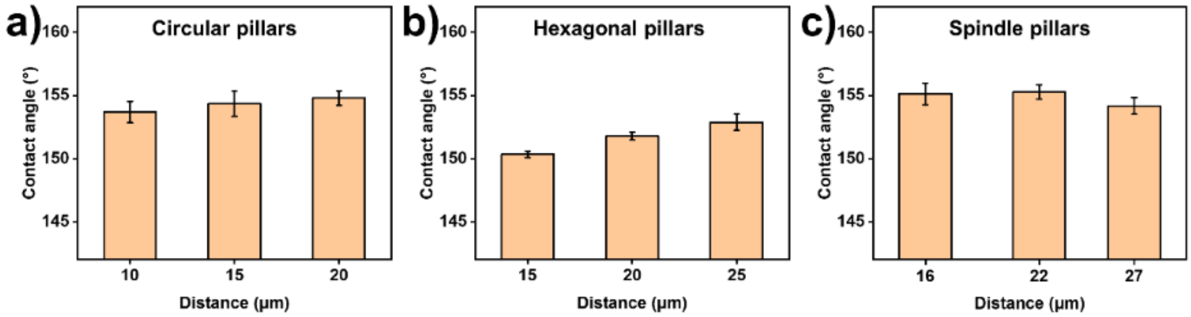

Figure S8. The superhydrophobicity of the micropillar-structured substrates with a square arrangement of circular micropillars with distances of 10, 15 and 20 μm a), a hexagonal arrangement of hexagonal micropillars with distances of 15, 20 and 25 μm b), a square arrangement of spindle micropillars with Y-axis distances of 16, 22 and 27 μm c).

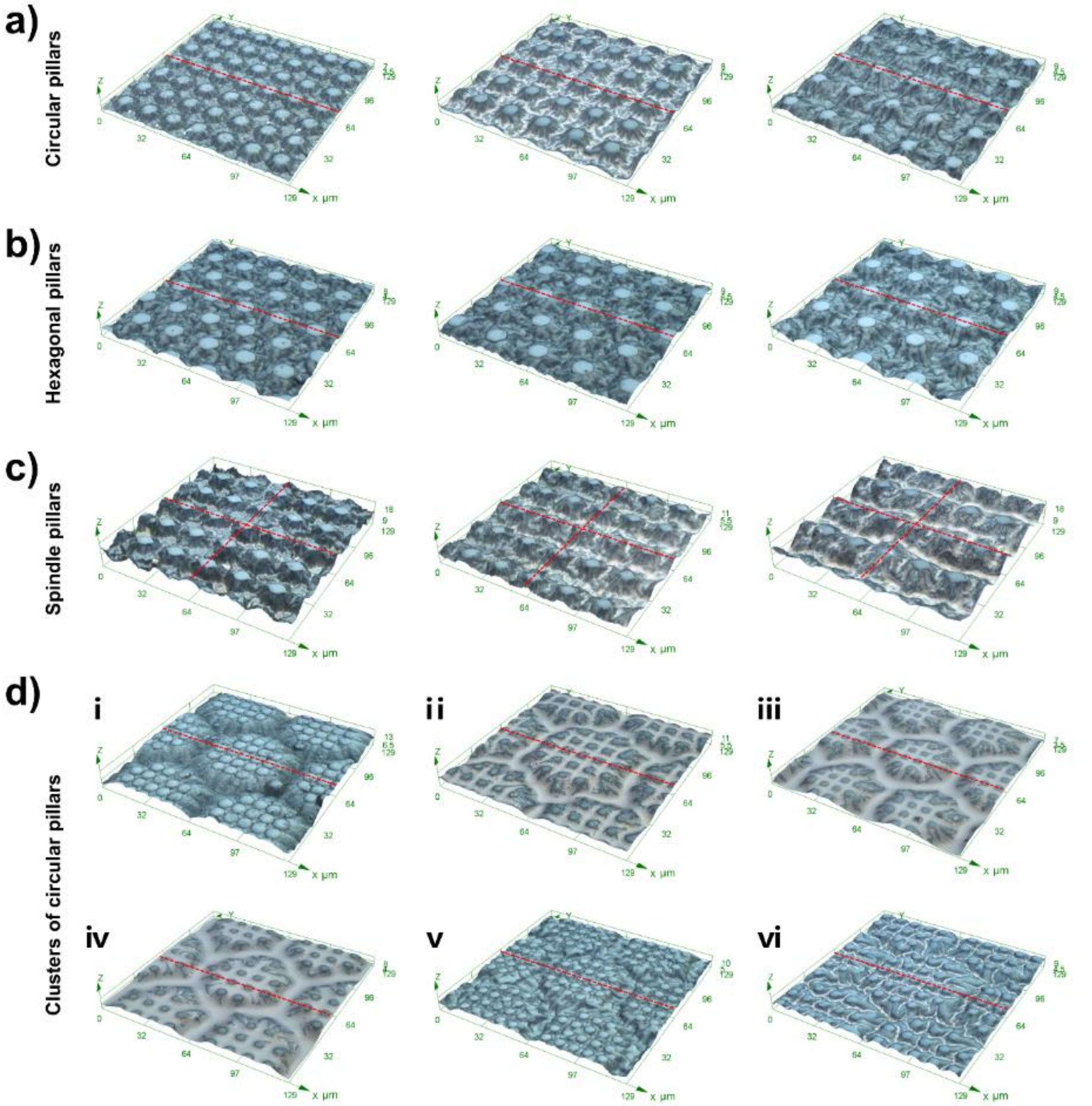

Figure S9. 3D laser-scanning microscopic images of McPCPFs with different base shapes in different arrangements constructed on micropillar-structured substrates with various geometrical parameters (e.g., shape and arrangement). 3D laser-scanning microscopic images of McPCPFs with a circular base in a square arrangement with diameter of 8 μm and distances of 10, 15 and 20 μm from left to right a), a hexagonal base in a hexagonal arrangement with side length of 5 μm and distances of 15, 20 and 25 μm from left to right b), an anisotropic spindle base in a square arrangement with X-axis length of 10 μm and Y-axis length of 7 μm, X-axis distance of 13 μm and Y-axis distances of 16, 22 and 27 μm from left

to right c), and a hierarchical circular base in a hexagonal arrangement with diameter of 3 μm, i) first-level distance of 26 μm and second-level distance of 6 μm, ii) first-level distance of 26 μm and second-level distance of 9 μm, iii) first-level distance of 26 μm and second-level distance of 6 μm, iv) first-level distance of 26 μm and second-level distance of 9 μm, v) first-level distance of 20 μm and second-level distance of 6 μm, vi) first-level distance of 20 μm and second-level distance of 9 μm d).

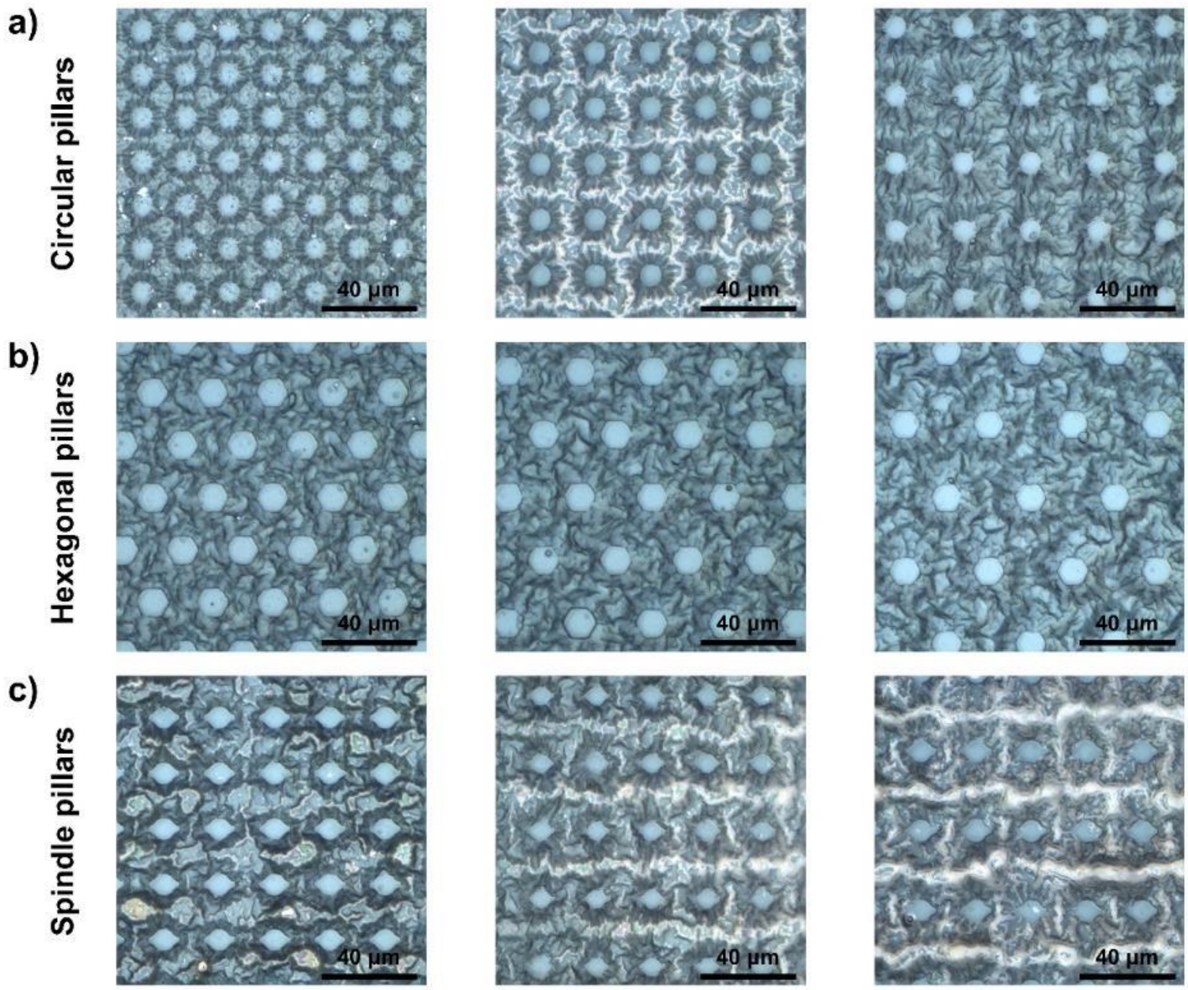

Figure S10. Plane laser-scanning microscopic images of McPCPFs with a circular base in a square arrangement with diameter of 8 µm and distances of 10, 15 and 20 µm from left to right a), a hexagonal base in a hexagonal arrangement with side length of 5 µm and distances of 15, 20 and 25 µm from left to right b), an anisotropic spindle base in a square arrangement with X-axis length 10 µm and Y-axis length 7 µm, X-axis distance of 13 µm and Y-axis distances of 16, 22 and 27 µm from left to right c).

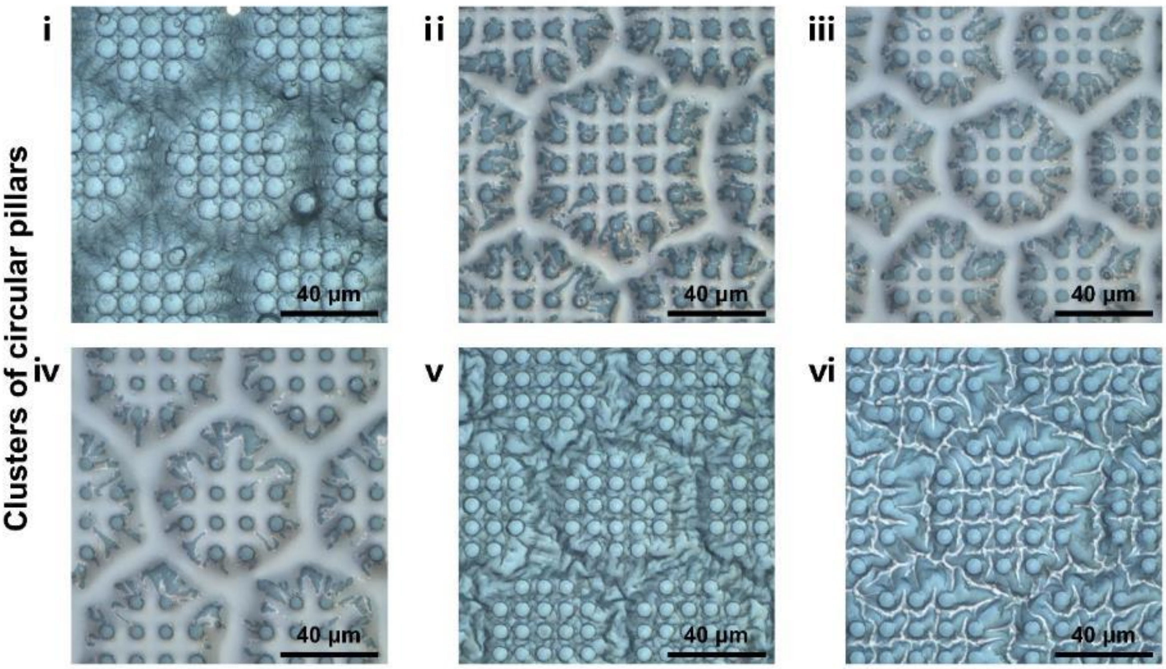

Figure S11. Plane laser-scanning microscopic images of McPCPFs with a hierarchical circular base in a hexagonal arrangement with diameter of 3 µm, i) first-level distance of 26 µm and second-level distance of 6 µm, ii) first-level distance of 26 µm and second-level distance of 9 µm, iii) first-level distance of 26 µm and second-level distance of 6 µm, iv) first-level distance of 26 µm and second-level distance of 9 µm, v) first-level distance of 20 µm and second-level distance of 6 µm, vi) first-level distance of 20 µm and second-level distance of 9 µm.

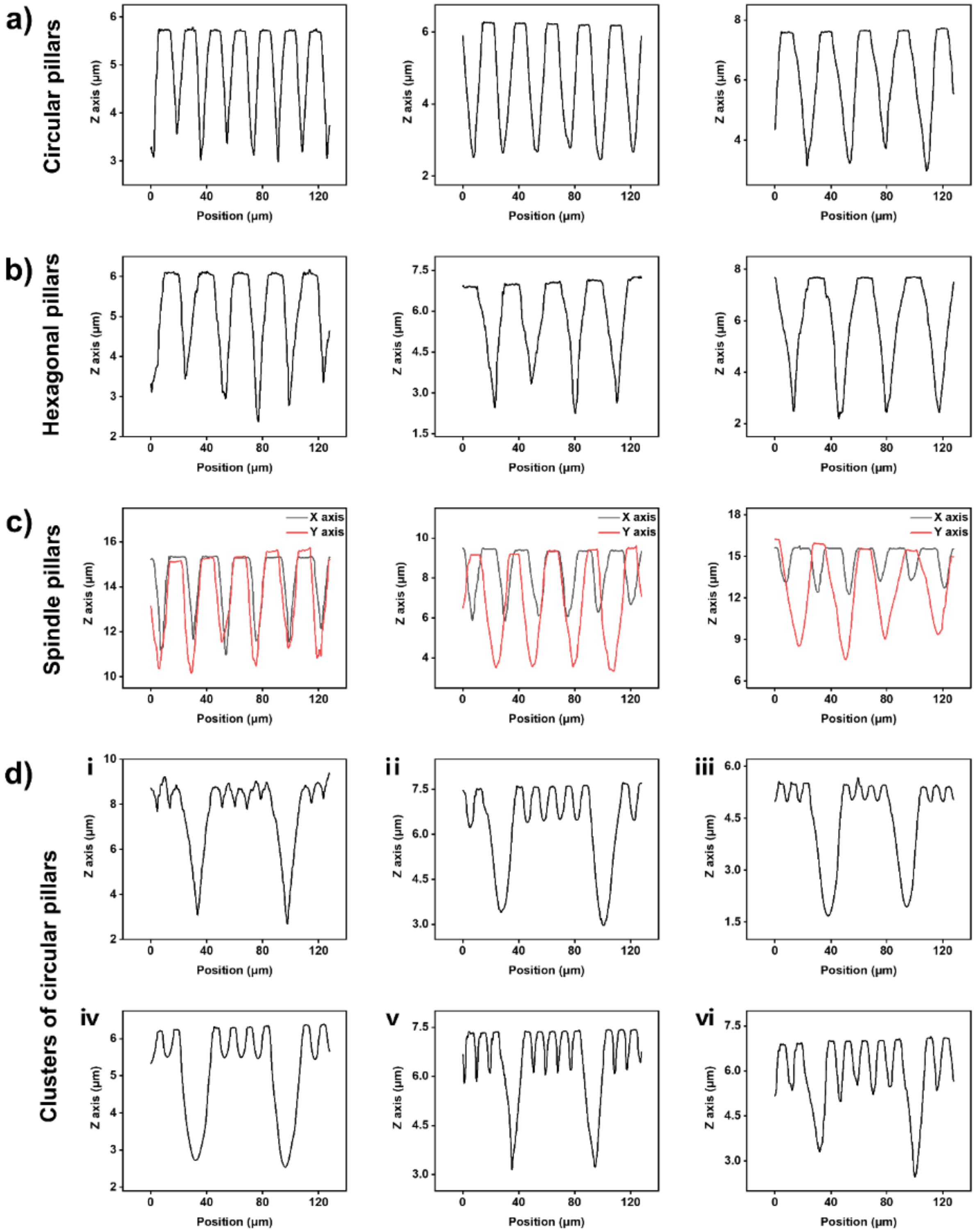

Figure S12. The corresponding profiles along the red dotted lines of McPCPFs with different base shapes in different arrangements as shown in Figure S9, including a circular base in a square arrangement a), a hexagonal base in a hexagonal arrangement b), an anisotropic spindle base in a square arrangement c), and a hierarchical circular base in a hexagonal

arrangement d).

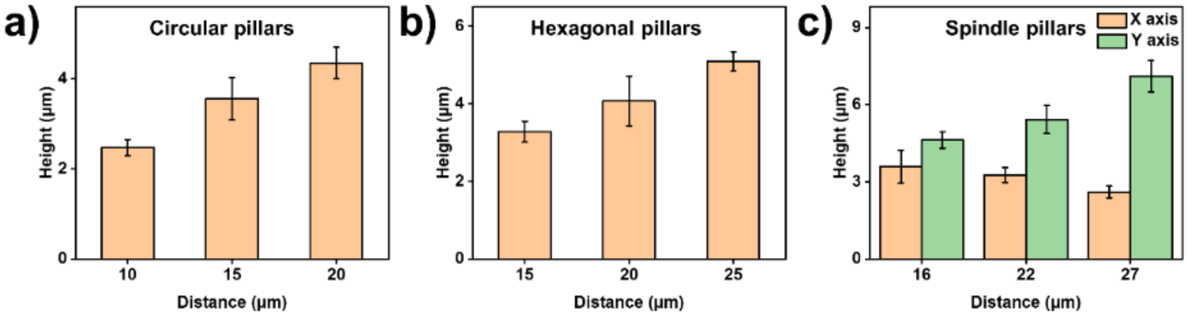

Figure S13. The dependence of the height of the microcavities with different base shapes in different arrangements on the micropillar distance. With the increase of the micropillar distance, the height of the microcavities with a circular base in a square arrangement a) and a hexagonal base in a hexagonal arrangement b) will increase. With the increase of the Y-axis distance, the height of the microcavities with a spindle base in a square arrangement will increase in the Y-axis direction and decrease in the X-axis direction c).

Table 4. The wettability and geometrical parameters of micropillar-structured substrates with a hexagonal arrangement of circular micropillar clusters, as well as the height of the hierarchical microcavities.

**Substrate serial number**	**i**	**ii**	**iii**	**iv**	**v**	**vi**
**Numbers of micropillars per cluster (N)**	**21**	**21**	**12**	**12**	**21**	**21**
**Diameter (d) (µm)**	**3**	**3**	**3**	**3**	**3**	**3**
**First-level distance between micropillar clusters (D_F) (µm)**	**26**	**26**	**26**	**26**	**20**	**20**
**Second-level distance between micropillars (D_S) (µm)**	**6**	**9**	**6**	**9**	**6**	**9**
**Contact angle (°)**	**155.7 ± 0.9**	**154.4 ± 0.9**	**156.1 ± 0.2**	**155.4 ± 1.2**	**152.8 ± 0.5**	**153.4 ± 1.1**
**First-level height (H_F) (µm)**	**5.93 ± 0.28**	**4.54 ± 0.4**	**3.63 ± 0.21**	**3.68 ± 0.15**	**4.02 ± 0.1**	**3.89 ± 0.08**
**Second-level height (H_S) (µm)**	**0.84 ± 0.16**	**1.46 ± 0.48**	**0.45 ± 0.05**	**0.81 ± 0.07**	**1.26 ± 0.03**	**1.64 ± 0.08**

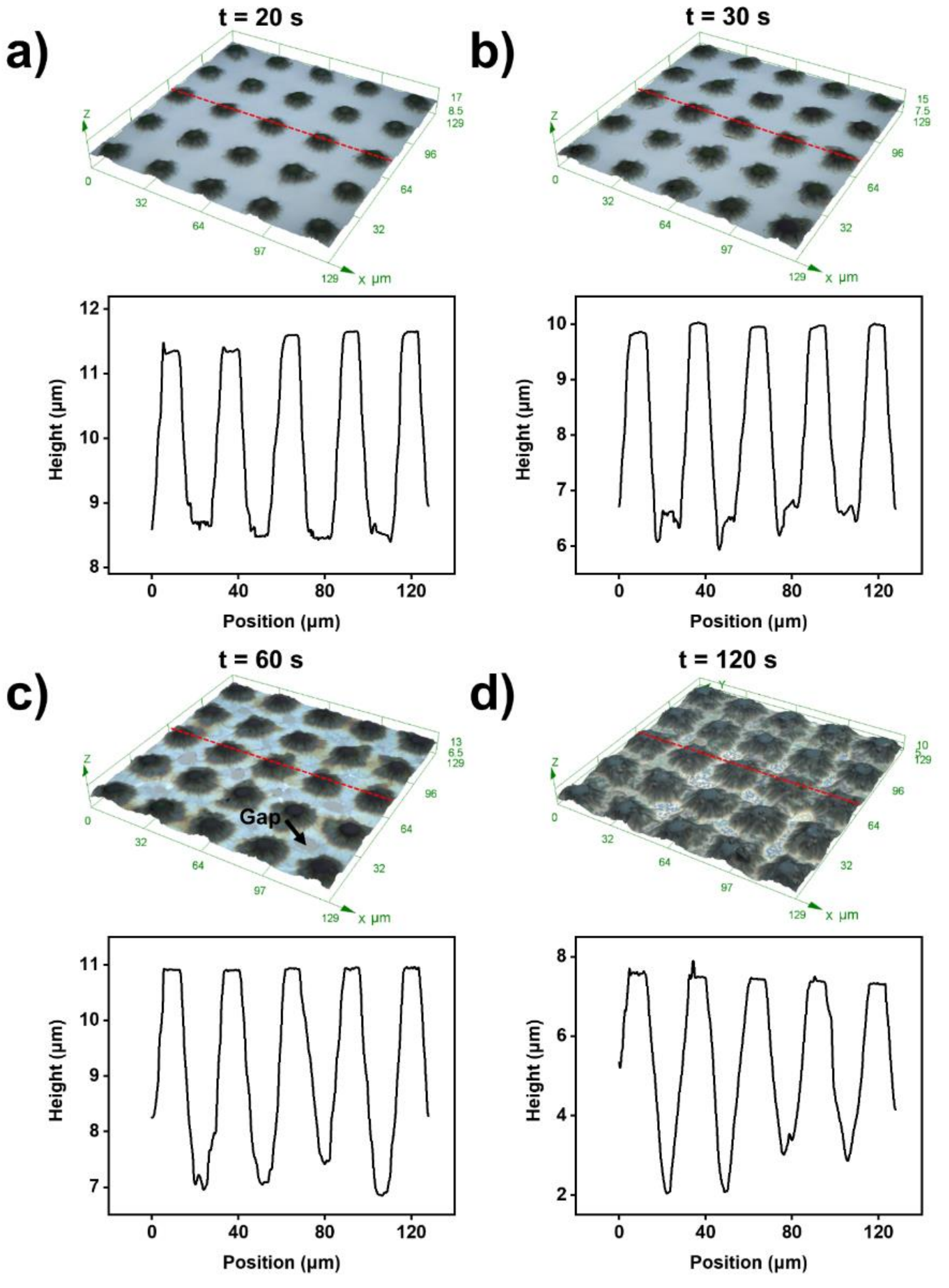

Figure S14. 3D laser-scanning microscopic images and the corresponding profiles along the red dotted lines of McPCPFs with a circular base in a square arrangement on micropillar-structured substrates in diameter of 8 μm and distance of 20 μm at polymerization times of 20 s a), 30 s b), 60 s c) and 120 s d).

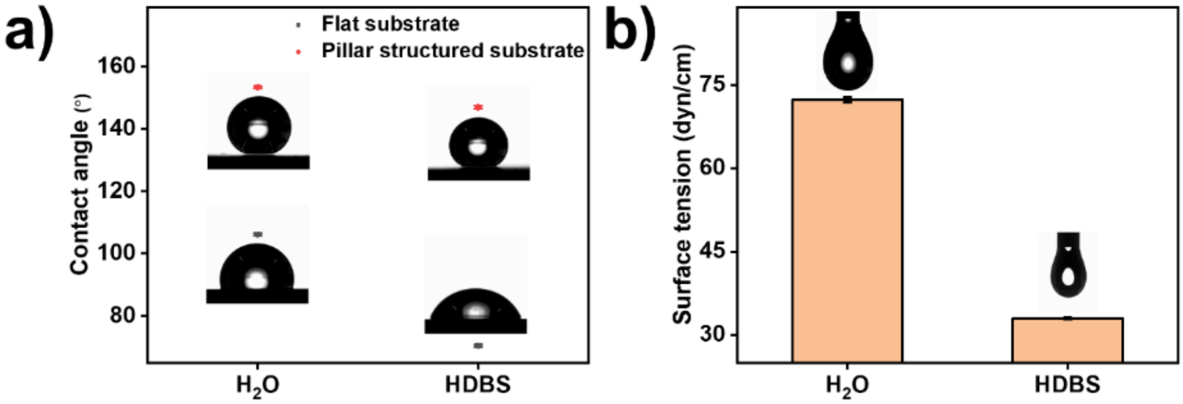

Figure S15. The contact angles and surface tension of water and the electrolyte solution with 0.01 M HDBS. a) The contact angles of the flat and micropillar-structured substrates with a square arrangement of circular micropillars in diameter of 8 μm and distance of 20 μm to water and the electrolyte solution. b) The surface tension of water and the electrolyte solution.

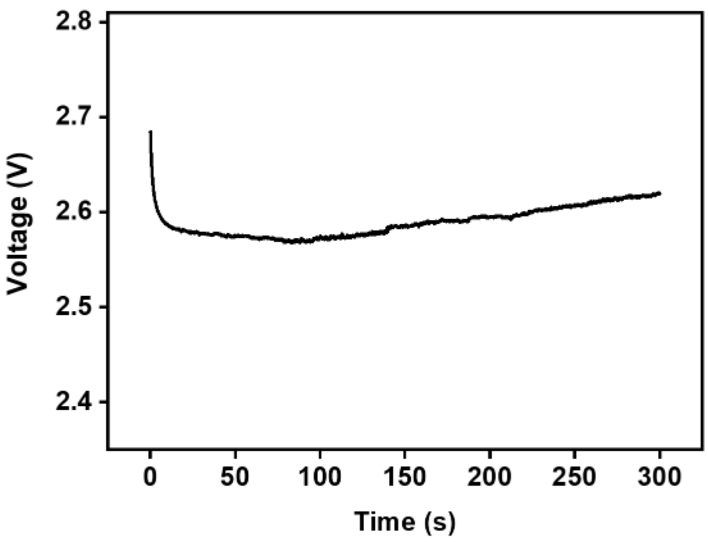

Figure S16. The voltage versus polymerization time curve of McPCPFs with polymerization time of 300 s.

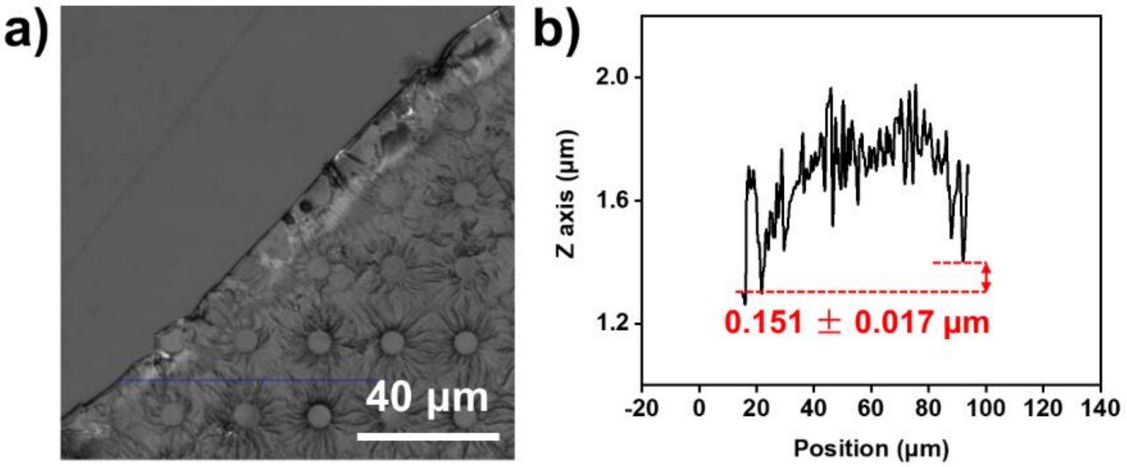

Figure S17. The plane laser-scanning microscopic image a) of a McPCPF transferred to a flat silicon substrate after polymerization for 300 s and the corresponding profile along the blue line b), indicating a thickness of 0.151 ± 0.017 μm.

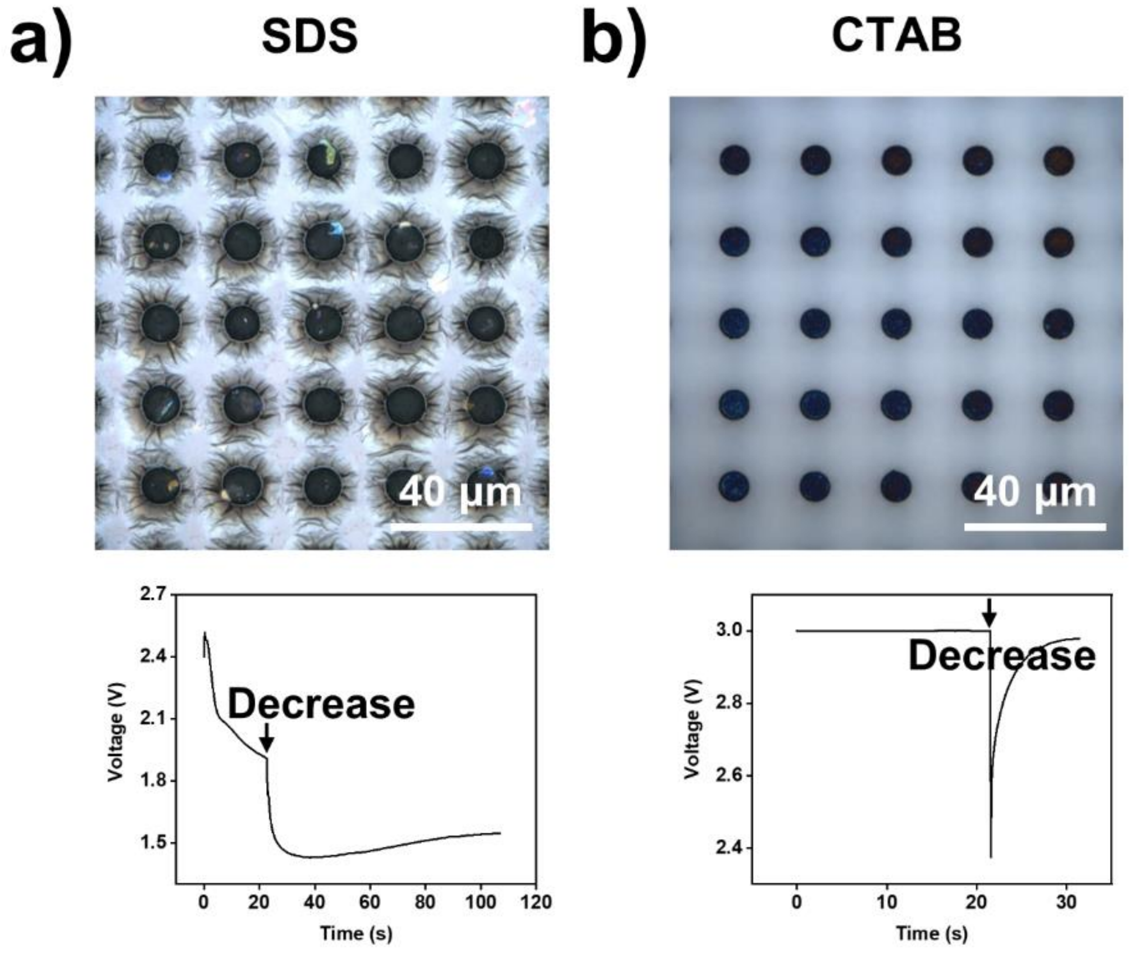

Figure S18. The plane laser-scanning microscopic images (top) and the corresponding voltage versus polymerization time curves (bottom) of patterned PPy polymerized on micropillar-structured substrates using electrolyte droplets consisting of 0.24 M pyrrole and 0.01 M sodium dodecyl sulfate (SDS) a), and 0.24 M pyrrole and 0.01 M hexadecyl trimethyl ammonium bromide (CTAB) b), respectively. The sharp decrease of the voltage indicated the penetration and filling of the electrolyte droplets.

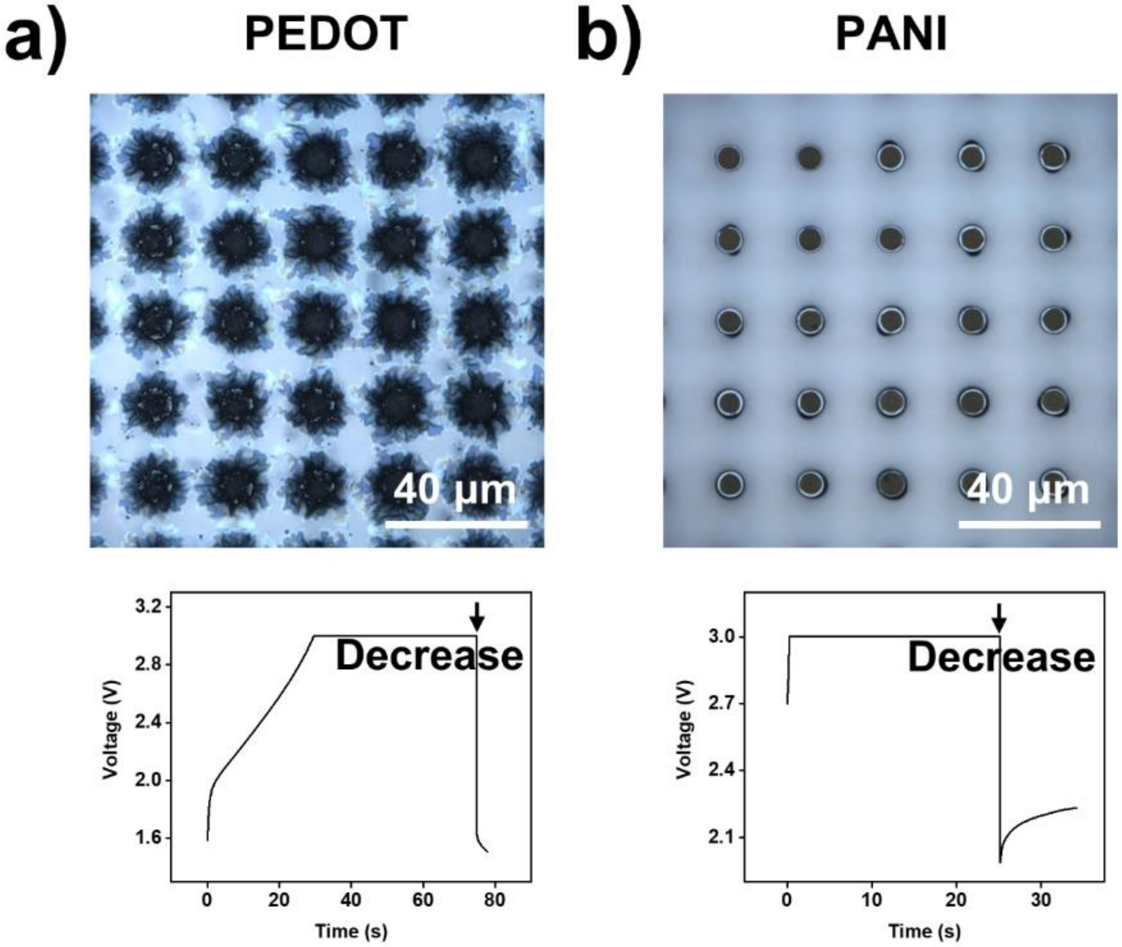

Figure S19. The plane laser-scanning microscopic images (top) and the corresponding voltage versus polymerization time curves (bottom) of patterned poly(3,4-ethylenedioxythiophene) (PEDOT) a) and polyaniline (PANI) b) polymerized on micropillar-structured substrates, respectively. The sharp decrease of the voltage indicated the penetration and filling of the electrolyte droplets.

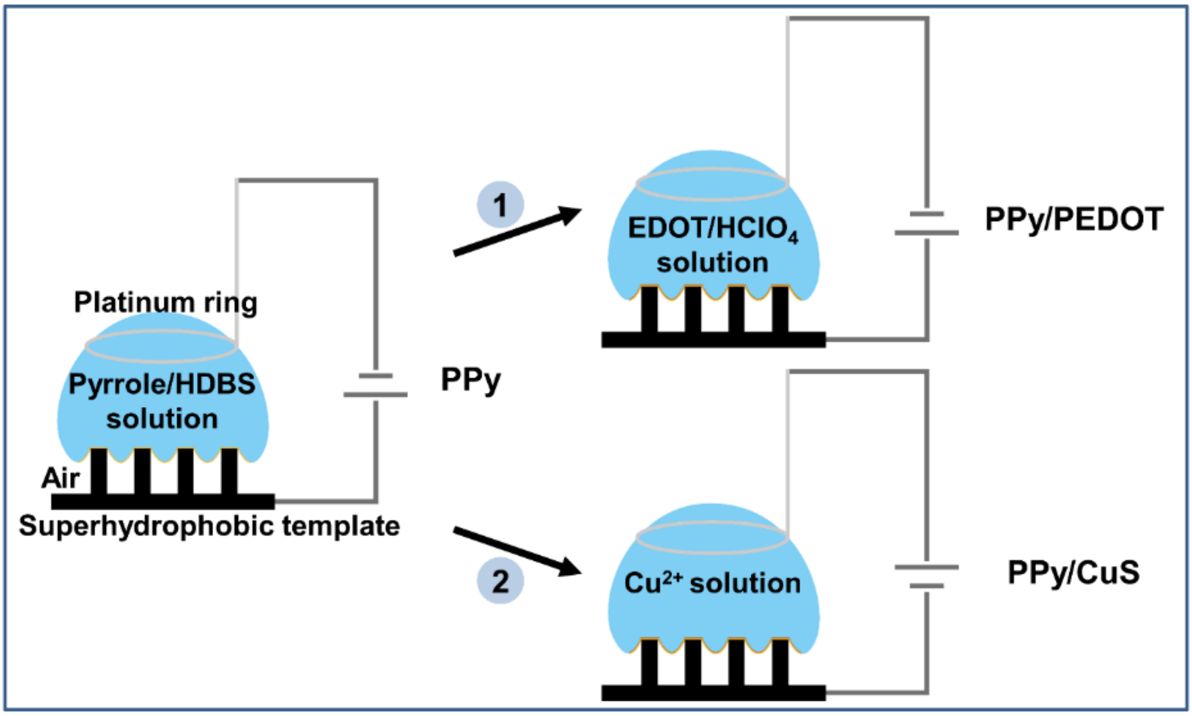

Figure S20. Schematic illustration of the fabrication process of the corresponding heterogeneous films *via* a two-step electrochemical reaction using the experimental setup. Process 1: The electropolymerization on the as-prepared PPy film is employed for obtaining the organic/organic heterogeneous film (i.e., PPy/PEDOT) with microcavities. Process 2: The electrochemical deposition on the as-prepared PPy film is used for obtaining the organic/inorganic heterogeneous film (i.e., PPy/CuS) with microcavities.

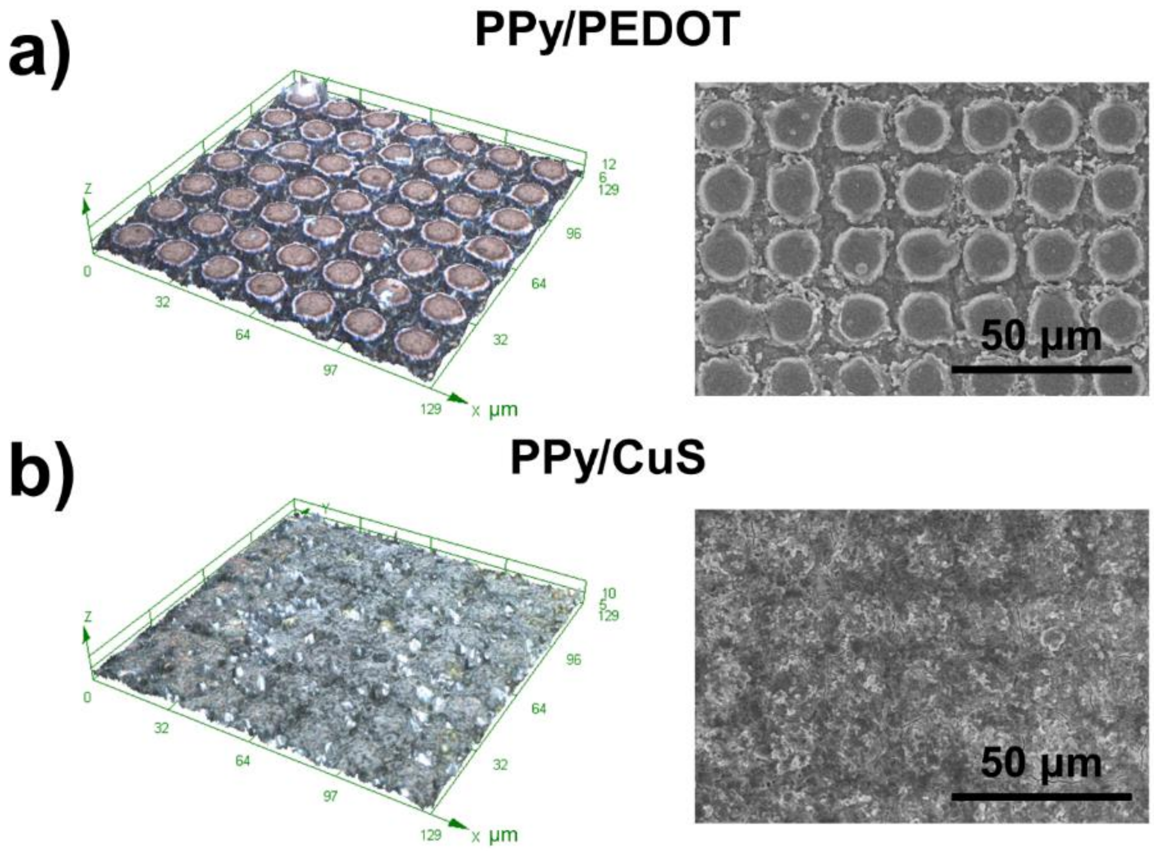

Figure S21. 3D laser-scanning microscopic images (left) and the corresponding ESEM images (right) of patterned PPy/PEDOT a) and PPy/CuS b) films *via* a two-step electrochemical reaction on micropillar-structured substrates, respectively.

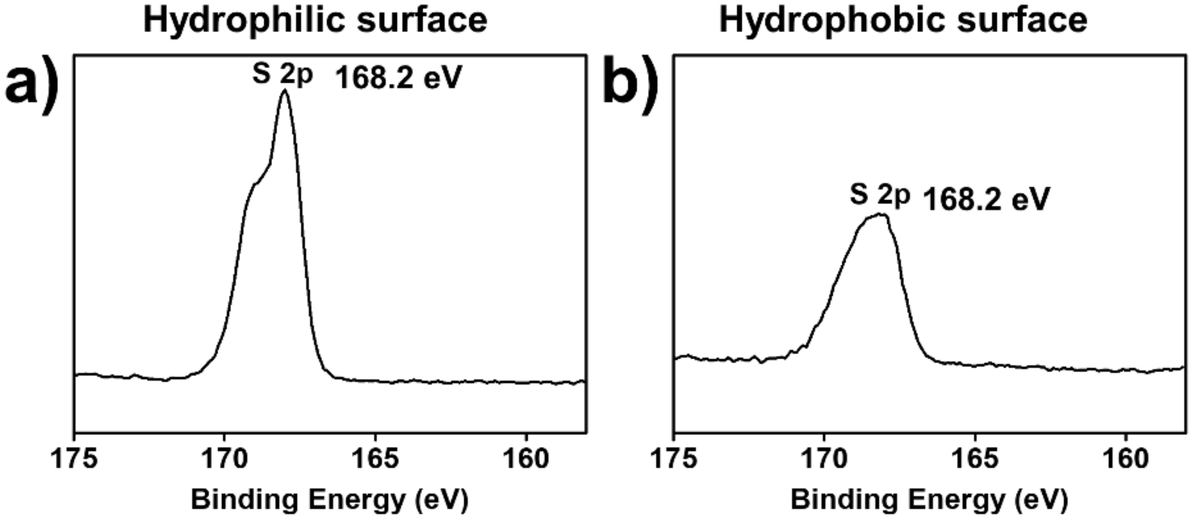

Figure S22. S 2p spectra of the hydrophilic surface a) and the hydrophobic surface b) of the McPCPFs, showing greater S 2p signal on the hydrophilic surface.

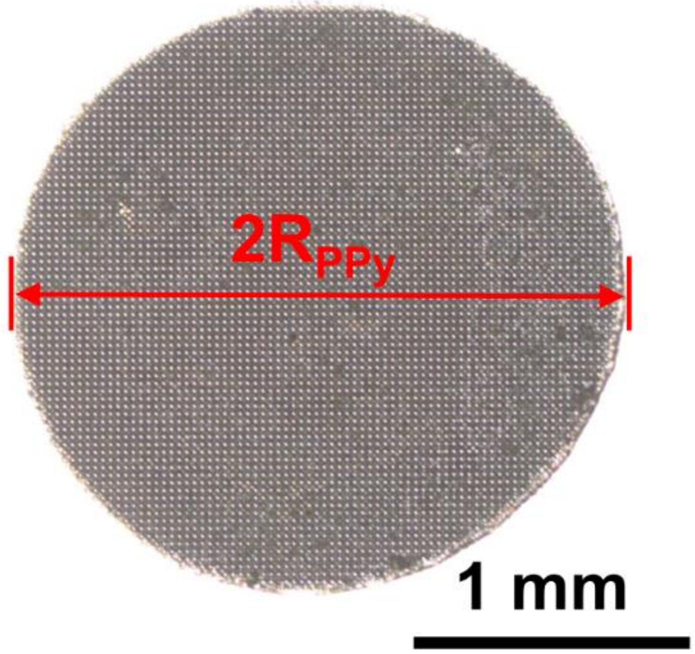

Figure S23. The microscopic image of the whole McPCPFs, showing an almost circular shape with a macroscopic diameter ($2R_{PPy}$) of 2.27 ± 0.19 mm.

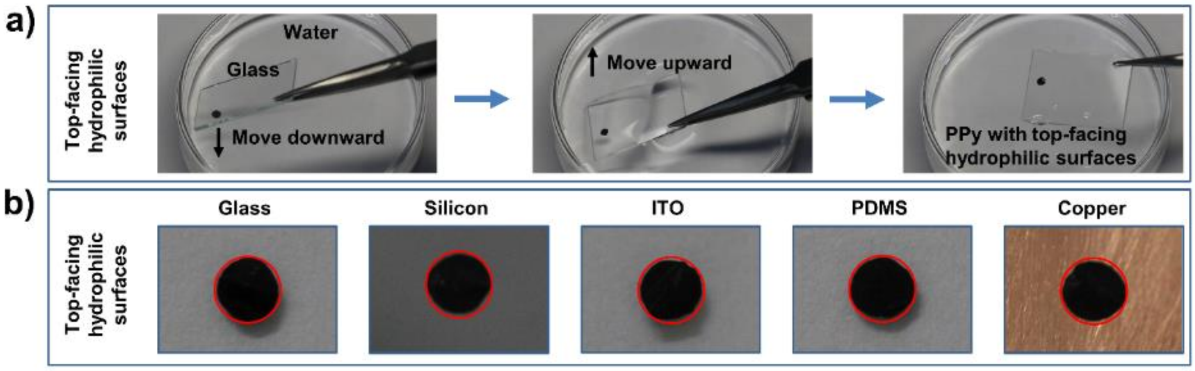

Figure S24. The nondestructive transfer process of the McPCPFs with the top-facing hydrophilic surface to various substrates. a) The transfer of the McPCPF with the top-facing hydrophilic surface to the glass surface, which includes moving the glass substrate downward to attach on the hydrophobic surface of the McPCPF on water and subsequently moving the glass substrate upward. b) The optical images of the McPCPFs with the top-facing hydrophilic surface on different substrates.

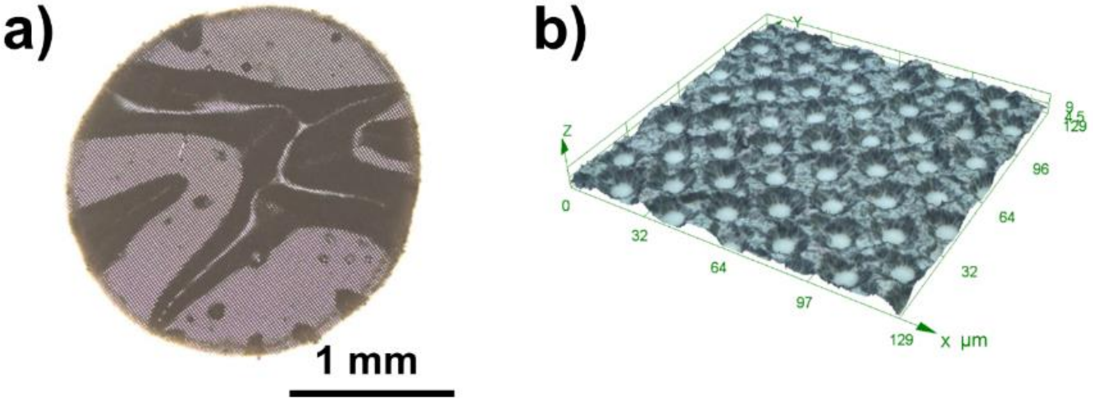

Figure S25. The high-resolution optical image a) and the 3D laser-scanning microscopic image b) for these McPCPFs after the transfer process.

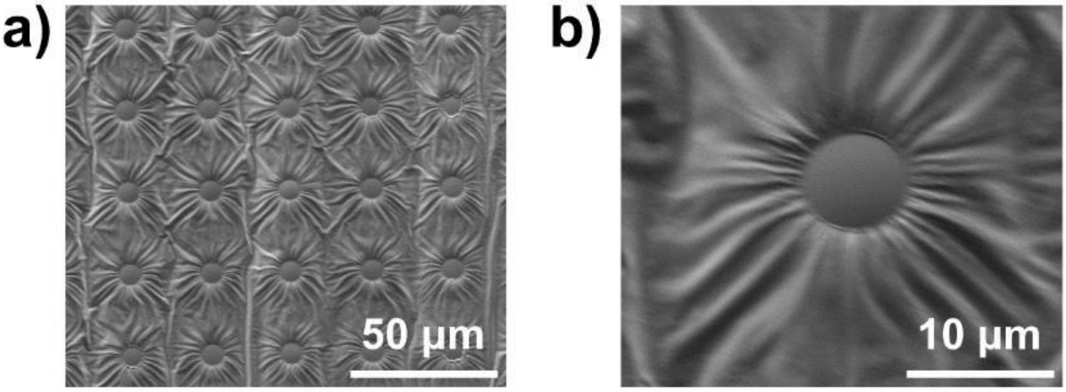

Figure S26. a) ESEM image of the McPCPFs with slight deformation on viscoelastic PDMS and b) enlarged ESEM image of an individual microcavity.

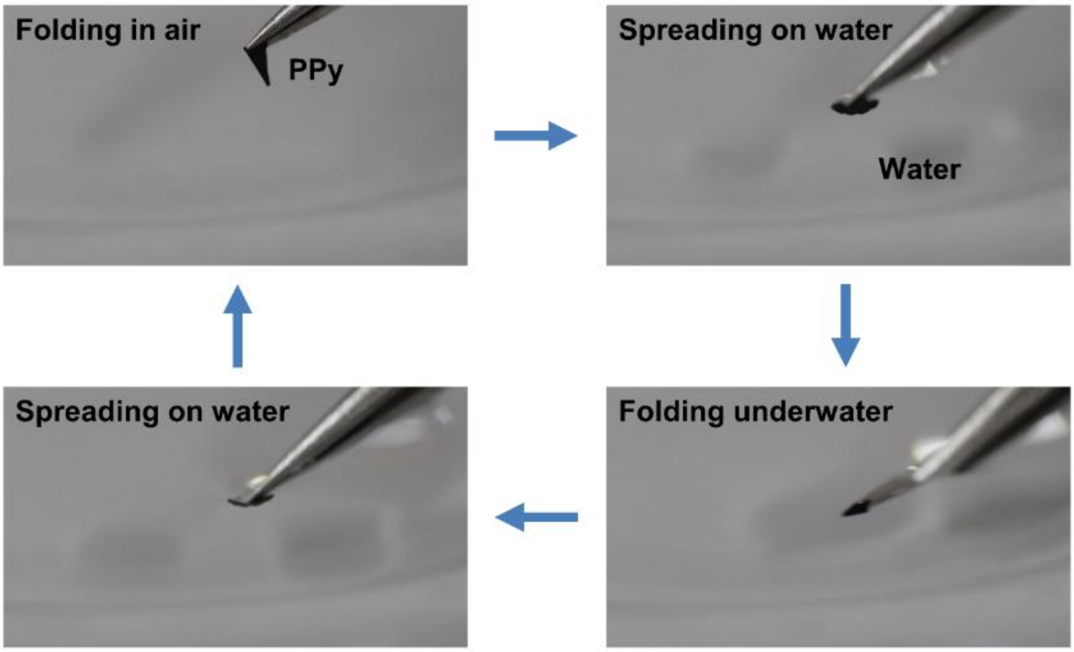

Figure S27. Thicker McPCPFs after polymerization for 600 s can withstand repeated folding and spreading during the process of entering into and out of the water.

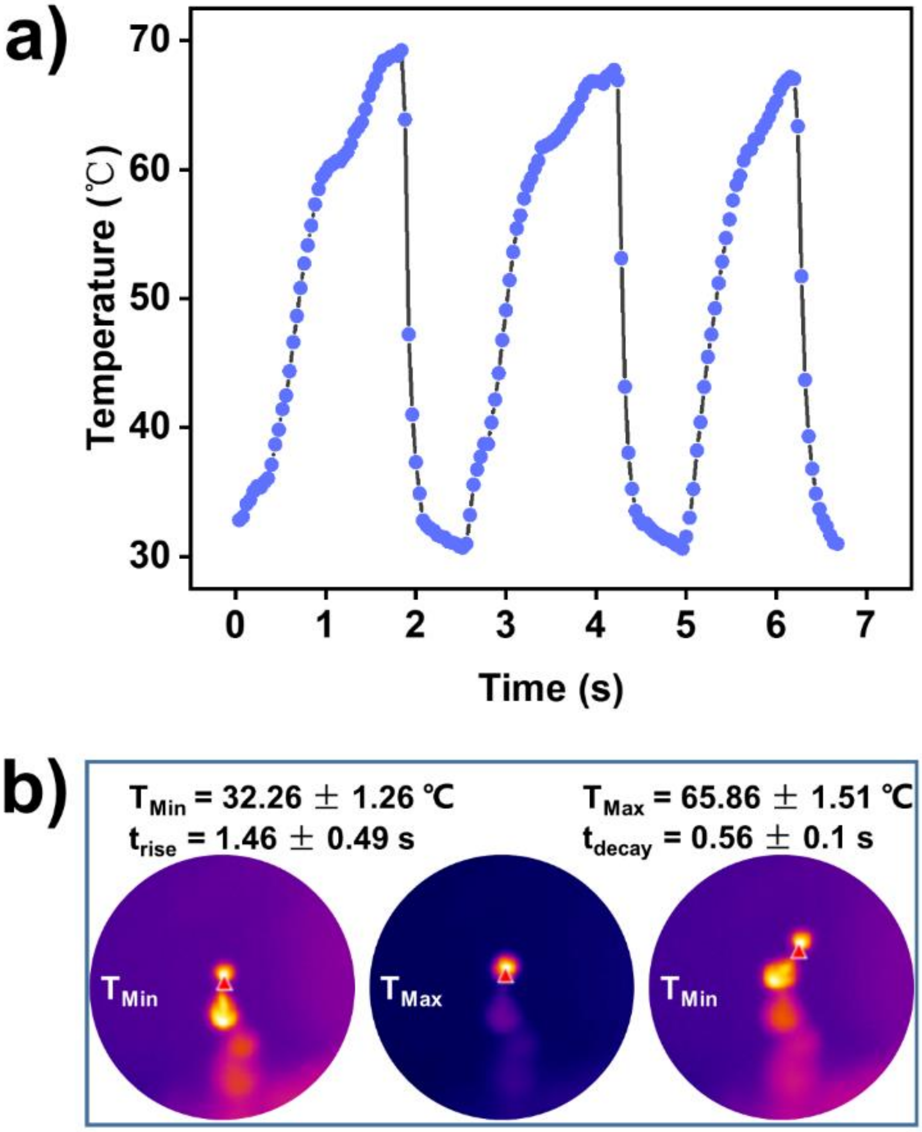

Figure S28. The temperature versus time curve of the flat conducting polymer films on DMSO surfaces during three repeated heating/cooling cycles a) and the corresponding infrared thermal images at T_{Min} and T_{Max} during one repeated heating/cooling cycle b).

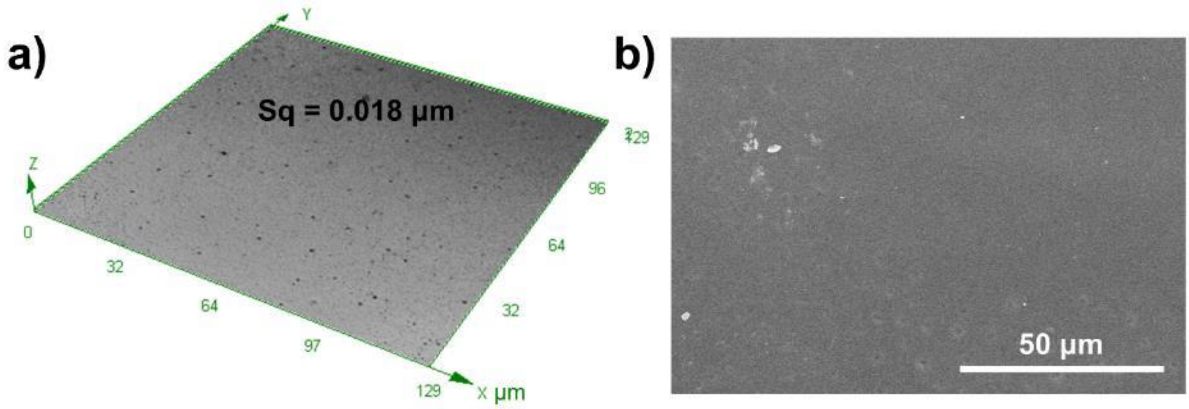

Figure S29. The 3D laser-scanning microscopic image of the flat conducting polymer film with the surface roughness of ~0.018 μm a) and the corresponding ESEM image b).

To demonstrate the application of the as-prepared McPCPFs, they were fabricated into the NH_3 detectors after the deposition of Au electrodes with the assistance of the copper mesh masks (Figure S30a). As shown in Figure S30b, the voltage versus current curve of the NH_3 detector without NH_3 was recorded firstly, which was linear and revealed the ohmic behavior of the McPCPF. Then, the real-time resistance change curve of the NH_3 detector with 300

ppm NH_3 was monitored during four repeated gas in/gas out cycles (Figure S30c). The result indicated that the resistance showed lower value of 5613 ± 29 Ω when there was no NH_3. Once 300 ppm NH_3 was applied to the detector for ~19 s (i.e., gas in), the resistance will increase to 6263 ± 89 Ω. When the 300 ppm NH_3 was removed (i.e., gas off), the resistance will return back to the initial value again for ~495 s. The corresponding real-time dynamic response can reach 11.54 ± 1.58% according to the following equation: $Response = \left({R_1}/{R_2} - 1\right) \times 100\%$, where R_1 and R_2 represent the real-time and the initial resistance of the NH_3 detector. Therefore, the NH_3 detectors consisting of the as-prepared McPCPFs can be employed to detect NH_3 reversibly and effectively, showing a response of 11.54 ± 1.58 % to 300 ppm NH_3 for ~19 s.

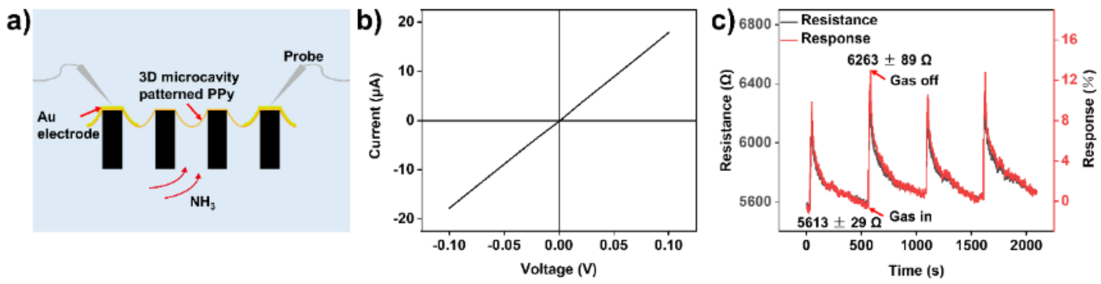

Figure S30. Demonstration of McPCPFs in detecting NH_3. a) Schematic of the NH_3 detector consisting of the McPCPF with a circular base in a square arrangement in diameter of 8 μm and distance of 10 μm. b) The voltage versus current curve of the NH_3 detector without NH_3, showing the ohmic behavior of the McPCPF. c) The real-time resistance change and response curves of the NH_3 detector with 300 ppm NH_3 during four repeated gas in/gas out cycles.
